# Scavenging Circulating Mitochondrial DNA as a Potential Therapeutic Option for Multiple Organ Dysfunction in Trauma Hemorrhage

**DOI:** 10.3389/fimmu.2018.00891

**Published:** 2018-05-08

**Authors:** Andrew Aswani, Joanna Manson, Kiyoshi Itagaki, Fausto Chiazza, Massimo Collino, Winston Liao Wupeng, Tze Khee Chan, W. S. Fred Wong, Carl J. Hauser, Chris Thiemermann, Karim Brohi

**Affiliations:** ^1^Department of Critical Care Medicine, Guy’s and St Thomas’ NHS Foundation Trust, London, United Kingdom; ^2^Centre for Trauma Sciences, Barts and The London School of Medicine and Dentistry, Queen Mary University of London, London, United Kingdom; ^3^Department of Surgery, Beth Israel Deaconess Medical Center, Harvard Medical School, Boston, MA, United States; ^4^Department of Drug Science and Technology, Università degli Studi di Torino, Turin, Italy; ^5^Department of Pharmacology and Immunology Program, National University Health System, Singapore, Singapore; ^6^Department of Translational Medicine and Therapeutics, Queen Mary University of London, London, United Kingdom

**Keywords:** trauma, mitochondrial DNA, damage-associated molecular patterns, nucleic acid scavenger, multiple organ dysfunction syndrome, trauma hemorrhage, Toll-like receptor-9, sterile inflammation

## Abstract

Trauma is a leading cause of death worldwide with 5.8 million deaths occurring yearly. Almost 40% of trauma deaths are due to bleeding and occur in the first few hours after injury. Of the remaining severely injured patients up to 25% develop a dysregulated immune response leading to multiple organ dysfunction syndrome (MODS). Despite improvements in trauma care, the morbidity and mortality of this condition remains very high. Massive traumatic injury can overwhelm endogenous homeostatic mechanisms even with prompt treatment. The underlying mechanisms driving MODS are also not fully elucidated. As a result, successful therapies for trauma-related MODS are lacking. Trauma causes tissue damage that releases a large number of endogenous damage-associated molecular patterns (DAMPs). Mitochondrial DAMPs released in trauma, such as mitochondrial DNA (mtDNA), could help to explain part of the immune response in trauma given the structural similarities between mitochondria and bacteria. MtDNA, like bacterial DNA, contains an abundance of highly stimulatory unmethylated CpG DNA motifs that signal through toll-like receptor-9 to produce inflammation. MtDNA has been shown to be highly damaging when injected into healthy animals causing acute organ injury to develop. Elevated circulating levels of mtDNA have been reported in trauma patients but an association with clinically meaningful outcomes has not been established in a large cohort. We aimed to determine whether mtDNA released after clinical trauma hemorrhage is sufficient for the development of MODS. Secondly, we aimed to determine the extent of mtDNA release with varying degrees of tissue injury and hemorrhagic shock in a clinically relevant rodent model. Our final aim was to determine whether neutralizing mtDNA with the nucleic acid scavenging polymer, hexadimethrine bromide (HDMBr), at a clinically relevant time point *in vivo* would reduce the severity of organ injury in this model. Conclusions: We have shown that the release of mtDNA is sufficient for the development of multiple organ injury. MtDNA concentrations likely peak at different points in the early postinjury phase dependent on the degree of isolated trauma vs combined trauma and hemorrhagic shock. HDMBr scavenging of circulating mtDNA (and nuclear DNA, nDNA) is associated with rescue from severe multiple organ injury in the animal model. This suggests that HDMBr could have utility in rescue from human trauma-induced MODS.

## Introduction

According to the latest data from the World Health Organization (1), trauma accounts for 10% of deaths and 16% of disabilities worldwide. Critically injured patients who survive their initial resuscitative phase are at a high risk of developing multiple organ dysfunction syndrome (MODS) (2). Isolated overwhelming tissue injury can lead to MODS but more commonly there is a combination of tissue injury and hemorrhagic shock. Half of MODS cases occur within 3 days post injury (3). The development of MODS is associated with complications such as sepsis, poor outcomes including death and high-resource utilization and health-care costs (4).

Traumatic injury can lead to a range of cellular injury including physical destruction, cell stress and frank necrosis, all of which can lead to the release of damage-associated molecular patterns (DAMPs) into the extracellular and vascular compartments (5–7). Mitochondrial DNA (mtDNA) has recently been identified as a DAMP and a potential important activator of the innate immune response to trauma (8). A number of diverse nucleic acid scavenging polymers (NASPs) have recently been evaluated *in vitro* and *in vivo* (9). Hexadimethrine bromide (HDMBr) is a NASP that has been shown to prevent the activation of endosomal toll-like receptors (TLRs) in a CpG DNA dependent toxic shock murine model and improve survival (10). If mtDNA is critical to the development of MODS, then the administration of a nucleic acid scavenging agent such as HDMBr may be a therapeutic opportunity in critically injured trauma patients.

The overall objective of this study was to understand the association of mtDNA release in trauma with the development of MODS and its potential for therapeutic modulation. We first aimed to determine whether mtDNA released after clinical trauma hemorrhage is sufficient for the development of MODS. We then aimed to determine the extent of mtDNA release with varying degrees of tissue injury and hemorrhagic shock in a clinically relevant rodent model. Our final aim was to determine whether neutralizing mtDNA with the NASP, HDMBr, at a clinically relevant time point *in vivo* would reduce the severity of organ injury in this model.

## Materials and Methods

### Human Study

The Royal London Hospital (RLH) is a busy, urban Major Trauma Centre and home to The London Air Ambulance. Trauma research is conducted at the RLH by The Barts Centre for Trauma Sciences (C4TS), Queen Mary University, London, who have been recruiting to a prospective, observational cohort study called the Activation of Coagulation and Inflammation in Trauma 2 (ACIT2) since 2008, to investigate the host response to traumatic injury.

Trauma patients are recruited on admission to the emergency department if they present within 2 h of injury. Blood samples are drawn on admission, 24 (±1 h) and 72 h and participants are seen daily until death or discharge. Written consent is obtained from all subjects although, if incapacitated, temporary consent can initially be obtained from a legally appointed representative (LAR). The study has approval from the National Health Service Research Ethics committee (REC): 07/Q0603/29. At the time of this clinical study, 367 patients had been enrolled in to ACIT2. From this biobank, 140 patients were selected for mtDNA measurement. The injury profile of the cohort was characterized using categories of Injury Severity Score (ISS) 0–4, 5–15, 16–25, and >25 and base deficit (BD) on admission −2 to 2, 2–6, and >6. Patients were randomly selected from each category, to achieve a balanced population for study. Control patients were defined as ISS 0–4 and no shock (BD −2 to 2 mEq/l). Therefore, our controls were part of the overall cohort and represent very minimally injured patients as opposed to healthy uninjured controls. MODS was defined using the Sequential Organ Failure Score (SOFA) as a score of ≥5 on at least 2 consecutive days, 48 h, or more following admission. One set of blank readings was subsequently excluded from analysis.

### Animal Study

#### Ethics Statement

All experiments were carried out using male Wistar rats (Charles River, UK) weighing between 280 and 350 g. Animals received a standard diet and free access to water during a 7-day adaptation period after transport into the laboratory from the supplier. This was performed in accordance with Home Office Guidance in the Operation of the Animals (Scientific Procedures) Act 1986 and the Guiding Principles in the Care and Use of Animals published by the American Physiological Society.

#### Anesthesia

All animals were anesthetized using intraperitoneal (i.p.) injections of sodium thiopentone, a barbiturate anesthetic agent, at a dose of 120 mg/kg (Merial Animal Health, UK). Small supplementary injections of thiopentone were administered intravenously during the course of the experiment as required.

#### Temperature Control

Animals were then placed supine onto a thermostatically controlled heating mat (Harvard Apparatus, UK). Body temperature was maintained at 38 ± 1°C with a temperature feedback *via* a rectal temperature probe connected to the homeothermic blanket. Desk lamps provided extra heat as required during particularly hypothermic phases such as controlled hemorrhage.

#### Airway and Ventilation

Airway patency and spontaneous respiration were facilitated by a tracheotomy and insertion of 2-cm length of polyethylene tubing (ID 1.67 mm, Portex, UK), approximately 5 mm into the trachea where it was secured with nylon sutures.

#### Arterial Catheterization

The left carotid artery was cannulated under direct vision with PE50 tubing (ID 0.58 mm, Portex, UK) and attached to a pressure transducer (AD Instruments, UK) and connected to a data acquisition system (Powerlab 8/35, AD Instruments, UK) for the measurement of mean arterial blood pressure (MAP) and heart rate (HR). The arterial catheter was primed with heparinized saline at a concentration of 50 IU/ml to prevent clot formation.

#### Venous Catheterization

The right jugular vein was cannulated under direct vision with PE25 tubing (ID 0.40 mm, Portex, UK) for the administration of fluid, blood or therapeutic agent. After baseline, instrumentation was completed, and animals were allowed to stabilize for 15 min before the next phase commenced.

#### Induced Traumatic Injury

1. Closed lower limb midshaft fractures. One or both tibias and fibulas were fractured manually, taking care not to break the overlying skin and tissue.
2. A 4-cm midline laparotomy. After hair removal and skin sterilization with 70% isopropyl alcohol wipes (Molnlycke Healthcare, UK), a midline incision was made without bowel manipulation. The wound was closed within 5 min in one layer with interrupted surgical 4/0 sutures (Prolene, Ethicon, UK). Highly standardized elements of both the trauma and hemorrhage phases were required to minimize variation and to allow the smallest number of animals to be used. Intentional or accidental bowel manipulation during laparotomy, for example, has been suggested as a driver for increased inflammation, remote organ injury and increased model severity (11, 12).
3. Bilateral lower limb muscle crush. Hemostatic forceps were applied to the upper musculature of each lower limb and clamped down maximally for 10 s.
4. Limb fracture hematoma, i.p. hematoma and laparotomy wound hematoma were excluded at necropsy.

#### Hemorrhagic Shock Protocol

During right-sided jugular vein catheterization, PE 25 tubing was advanced gently into the right heart such that blood could be freely aspirated. Jugular catheter tip positioning was confirmed at necropsy. Hemorrhagic shock was induced *via* bleeding from the jugular catheter to achieve an MAP of 35 ± 5 mmHg within 10 min, at a rate no faster than 1 ml/min. Hemorrhage was continued for 20–35 min until 20–30% of the estimated blood volume had been removed. No resuscitation was delivered to the animal subsequently. The carotid arterial catheter was primed with a reduced concentration of heparinized saline (25 IU/ml) to maintain patency. If the MAP signal indicated a problem with catheter patency, every attempt was made to bleed back the catheter to remove any potential heparinized saline before flushing with plain saline of 20 µl (the dead space of a 150-mm length of PE25 tubing), then reconnecting to the heparinized saline system and finally, re-priming with 20-µl heparinized saline. Thus, systemic administration of heparin was minimized.

#### Therapeutic Agent Administration

At 15 min after the hemorrhage phase was completed, animals received either an intravenous bolus of saline 250 µl (0.9% NaCl, Baxter Healthcare, UK) or the NASP study drug (HDMBr, Sigma, UK) at a concentration of 1–4 mg/kg, dissolved in saline of 250 µl, both administered for 10 min.

#### Point of Care Tests

##### Lactate Measurement

A 20-µl blood sample was withdrawn and used for lactate measurement (Accutrend, Roche, UK).

##### Blood Gas Analysis

A 100-µl blood sample was aspirated into a heparin-coated (heparin fully expelled) syringe for blood gas analysis (Radiometer, UK).

#### Plasma Preparation

Terminal blood was collected into EDTA containing tubes (3 × 1.3 ml, Sarstedt, UK), gently inverted three times and immediately centrifuged at 200 *g* for 10 min at room temperature to remove the platelet-rich fraction. The plasma layer was then aspirated taking care not to disturb the cellular fraction and again centrifuged at 3,000 *g* for 15 min at 4°C, and then again at 3,000 *g* for 15 min at 4°C.

#### Biochemical Organ Function Analysis

Plasma samples were sent to a contract laboratory (Vetlab Services, Sussex, UK) for analysis within 24 h for urea, creatinine, alanine aminotransferase (ALT), aspartate aminotransferase (AST) and creatine kinase (CK). Renal dysfunction was quantified by the rise in urea (a marker of pre-renal renal impairment and/or increased catabolism) and creatinine (a marker of impaired glomerular filtration rate) (13). Liver injury was quantified by a rise in ALT (a specific marker of parenchymal damage) and also in AST (a nonspecific marker of liver injury, which is also raised in myocardial, renal and muscle necrosis) (14). Muscle injury was quantified by a rise in CK, which is also elevated in cardiac muscle injury and brain injury (15).

#### Lung Myeloperoxidase (MPO) Activity

Myeloperoxidase activity, used as an indicator of leukocyte accumulation into the lung, was determined as previously described (16). Briefly, samples were homogenized and centrifuged for 30 min at 13,000 *g* at 4°C. An aliquot of the supernatant was then allowed to react with a solution of 1.6 mM tetramethylbenzidine and 0.1 mM H_2_O_2_. The rate of change in absorbance was measured spectrophotometrically at 460 nm. MPO activity was defined as the quantity of enzyme degrading 1 µmol of peroxide per min at 37°C and was expressed in milliunits per gram of wet tissue. All compounds were from Sigma, MO, USA.

#### Plasma and Lung Cytokine Analysis

Commercial colorimetric rat ELISA kits for the measurement of plasma IL-1β, IL-6 and TNF-α were used (R&D Systems, UK). Rat HMGB1 (MyBioSource, USA) and TFAM (Cusabio, Japan) ELISA kits were also used. Plasma dilutions were none (neat) for all ELISA kits except IL-6 (1:2 dilution). Lung homogenates were tested for IL-6 concentration using a rat ELISA kit (Sigma, UK).

#### Extraction of Free Circulating DNA in Cell-Free Plasma

DNA was extracted from cell-free EDTA plasma with the QIAamp Blood Mini kit (Qiagen, UK). Frozen plasma was thawed over ice, vortexed for 5 s and then centrifuged at 1,600 *g* for 5 s. Qiagen Protease or proteinase K was used. DNA was eluted into 60-µl sterile water. DNA purity and yield was examined with the spectrophotometer (Nanodrop, Thermo Fisher Scientific, UK).

#### Real-Time Polymerase Chain Reaction (RT-PCR) to Measure Plasma Circulating mtDNA and nDNA

Primers for three rat mtDNA genes and one nDNA gene were used (Invitrogen, UK): Cytochrome B (Cyt B) TCCACTTCATCCTCCCATTC (Forward) CTGCGTCGGAGTTTAATCCT (Reverse); Cytochrome C oxidase subunit III (Cyto C III) ACATACCAAGGCCACCAAC (Forward) CAGAAAAATCCGGCAAAGAA (Reverse); NADH dehydrogenase CAATACCCCACCCCCTTATC (Forward) GAGGCTCATCCCGATCATAG (Reverse); Glyceraldehyde 3-phosphate dehydrogenase (GAPDH) GAAATCCCCTGGAGCTCTGT (Forward) CTGGCACCAGATGAAATGTG (Reverse). GAPDH is a nuclear gene that is expressed at high levels in most tissues and cells, and is considered a housekeeping gene. GAPDH is commonly used as a loading control for western blot and as a control for RT-PCR (17). However, this study was also concerned with nDNA concentrations *per se*. All primer sequences were specific for their targets and had no similarity with bacterial sequences on BLAST analysis. PCR reaction volume was 20 µl (containing 6-µl DNA) using SYBR Green Mastermix (Life Technologies, UK) and primers in a final concentration of 1.0 µM. The Rotorgene 6000 RT-PCR machine was used (ex-Corbett Life Science, currently Qiagen, UK) to perform 40 cycle PCR comprising 10 s hold at 95°C, 30 s annealing at 55°C, and 30-s extension at 72°C. Data analysis was performed on Corbett Life Science proprietary software. Absolute quantification of mtDNA was performed using serial dilutions of pure mtDNA extracted from rat liver to generate standard curves. NDNA was quantified using 1/Ct values relative to the change in sham levels.

#### Preparation of Pure mtDNA From Rat Liver

Mitochondria were isolated from rat liver either with the use of the Mitochondrial Isolation Kit (Sigma, UK). Liver was prepared in a sterile manner at 4°C. Liver was homogenized at 4°C with a 3-ml volume electric homogenizer for 30 s. DNA was extracted using the QIAamp DNA mini kit (Qiagen, UK) using proteinase K. Yield and purity were assessed spectrophotometrically.

#### Bacterial 16S rRNA PCR Screening of Plasma and Pure mtDNA Fractions

Bacterial screening of animal cell-free plasma and pure mtDNA fractions was performed using RT-PCR against bacterial 16S rRNA. NADK primers specific for 16S were used (Invitrogen, UK) and NADK probe modified with FAM-BHQ (black hole quencher) and shrimp nuclease (Affymetrix, UK) to remove contaminating bacteria present in reagents. PCR reaction volume was 10 µl comprising 2.9-µl molecular grade water (Ambion, Thermo Fisher Scientific, UK), 5-µl SSOFAST probe supermix (Bio-Rad, UK), 0.8-µl primers (F + R), 10-µM concentration, 0.2-µl probe, and 0.1-µl shrimp nuclease. CFX 96 RT-PCR machine with C1000 thermal cycler (Bio-Rad, UK) used. 40 cycles: 2 min at 95°C, 10 s at 61.4°C, 5 s at 95°C. *Enterococcus faecalis* standards were serially diluted 1:10 from 200 ng/µl to 20 fg/μl.

#### NASP Toxicity Experiments

Healthy controls were anesthetized and instrumented as described previously. NASP 2 or 4 mg/kg in 250-µl saline of 0.9% total volume was intravenously injected into animals for 10 min. Plasma and organs were sampled at 6 h.

#### Pure mtDNA Challenge in Healthy Animals

Healthy controls were anesthetized and instrumented as described previously. Pretreatment with NASP 2 mg/kg was followed by intravenous injection of pure mtDNA from 5% liver by weight (Sigma, UK). Pure mtDNA from either 3 or 5% liver was also intravenously injected alone into healthy instrumented animals. Plasma and organs were sampled at 6 h.

#### Lung Tissue Western Blot Analysis

The analysis was performed in association with Prof. Massimo Collino, Department of Drug Science and Technology, Turin University, Italy. Western blots were carried out as previously described (18). Proteins were separated by 8% sodium dodecyl sulfate-polyacrylamide gel electrophoresis and transferred to polyvinylidene difluoride membrane, which was then incubated with primary antibodies [rabbit anti-NF-κB p65, rabbit anti-STAT-3, and rabbit anti-phospho-signal transducer and activator of transcrip-tion (STAT)-3]. Blots were then incubated with a secondary antibody conjugated with horseradish peroxidase (HRP) (dilution 1:10,000) and developed using the ECL detection system. The immunoreactive bands were visualized by autoradiography and the density of the bands was evaluated densitometrically using Gel Pro^®^Analyzer 4.5, 2000 software (Media Cybernetics, Silver Spring, MD, USA). The membranes were stripped and incubated with β-actin monoclonal antibody (dilution 1:5,000) and subsequently with an anti-mouse antibody (dilution 1:10,000) to assess gel-loading homogeneity. Unless otherwise stated, all compounds were purchased from the Sigma-Aldrich, MO, USA. The BCA Protein Assay kit and SuperBlock blocking buffer were from Pierce Biotechnology (IL, USA). Antibodies were from New England Biolabs, UK. Luminol ECL was from Amersham (Buckinghamshire, UK).

#### Lung Histological Analysis

The rat lung was fixed in 10% formalin (Sigma) for 24 h, followed by embedment and 6-µm sectioning for hematoxylin & eosin (H&E) staining. Samples were dehydrated using graded ethanol, embedded in paraffin wax, and cut into sections using a Leica rotary microtome (thickness, 6 µm). Sections were deparaffinized with xylene, stained with Gills hematoxylin, and washed. Sections were then subsequently counterstained with 1% eosin, dehydrated with ethanol, and cleared with Neo-Clear (Darmstadt, Germany) before mounting using HistoMount (Atlanta, GA, USA). Sections were analyzed using a Leica DM2000 upright microscope (Wetzlar, Germany). The entire H&E-stained section was evaluated at low magnification (5× objective) for inflammatory cell infiltration. A 4-point scoring scale of cell infiltration was used to determine the grade of lung inflammation: 0 = normal; 1 = mild; 2 = intermediate; 3 = severe (19). Features examined were inflammatory cell infiltration, pulmonary congestion, and thickening of the alveolar septa. A total of 10 fields were evaluated randomly for each sample. The score for each group was the average score for all samples in the group. Quantitative analysis was performed in a blinded way.

#### Lung Immunohistochemistry Analysis

Paraffin-embedded sections were deparaffinized with histo-clear/ethanol and rehydrated. Antigen retrieval was made in sodium citrate buffer (10 mM, pH 6.0) at a sub-boiling temperature for 10 min, followed by 30-min cooling on bench top. After incubation with 3% hydrogen peroxide to remove endogenous peroxidase activity, the slides were washed with PBS and blocked with 5% normal serum for 1 h. The sections were immunostained with anti-rat cleaved caspase-3 (1:1,000, Cell Signaling Technology, Danvers, MA, USA) and anti-rat nitrotyrosine (1:1,000, EMD Millipore, Temecula, CA, USA) at 4°C overnight. After washing with PBST, the slides were incubated with a biotinylated secondary antibody (Vector labs, Burlingame, CA, USA), followed by streptavidin-HRP (Vector labs). The bound antibodies were developed by ImmPACT™ DAB peroxidase substrate kit (Vector labs). The cleaved caspase-3^+^ cell number was quantified by counting positively stained cells in 20 randomly selected fields under 60× objective.

#### Statistical Analysis

All statistical analyses were performed using Graphpad Prism version 6. Parametric data were analyzed using Student’s *t*-test; multiple groups using ANOVA with Dunnett’s/Tukey’s comparisons as appropriate. Non-parametric data were analyzed using chi-squared tests. Mean values with SEM were quoted throughout unless otherwise stated.

## Results

### Human Study

This human study examined mtDNA levels in 139 trauma patients, and demonstrated that patients who developed MODS had significantly higher concentrations of mtDNA in their circulation at only 2 h following injury.

**Figure 1:**
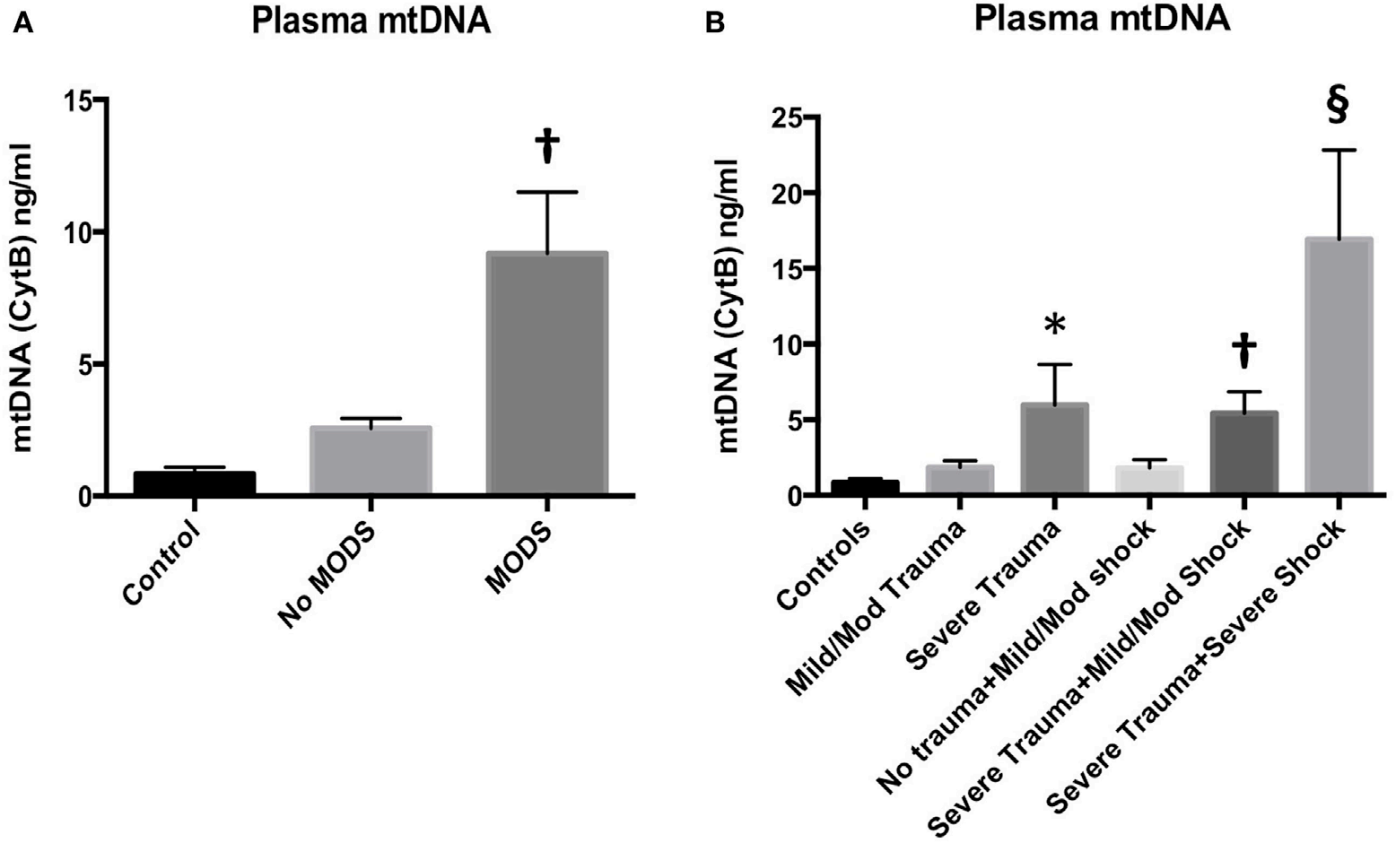
**(A)** (left) Plasma mitochondrial DNA (mtDNA) concentration at 2 h from injury is associated with the development of multiple organ dysfunction syndrome (MODS) in injured trauma patients. Injured patients who developed MODS (*n* = 27) had higher concentrations of mtDNA (as measured by cytochrome B concentration) in their peripheral blood than injured patients who did not develop MODS (*n* = 85). Control subjects had an Injury Severity Score (ISS) of 0–4 and normal base excess (*n* = 16) mean (95% CI): 0.9 (0.4–1.3) ng/ml, no MODS: 2.6 (1.8–3.3) ng/ml, MODS 9.2 (4.6–13.7) ng/ml. Bar graphs indicate mean values with SEM. † denotes *p* < 0.01 when no MODS and MODS groups were compared with a *t*-test. DNA was extracted from cell-free plasma and mtDNA was measured using real-time polymerase chain reaction with cytochrome B as the target gene. **(B)** (right) Plasma mtDNA concentration in patients at admission with increasing tissue injury and shock from left to right. mtDNA levels demonstrated a dose-dependent relationship with ISS. Controls (ISS 0–4 and normal base excess, *n* = 16) mean ± SEM mtDNA 0.8471 ± 0.2525 ng/ml, mild/moderate trauma (ISS 5–24, normal BE, *n* = 34), 1.850 ± 0.4266, and severe trauma (ISS ≥ 25, normal BE, *n* = 16) 5.960 ± 2.691. * denotes *p* < 0.05 vs controls, ANOVA/Dunnett’s. Isolated shock (ISS 0–4, BE −2.1 to −10, *n* = 11) did not significantly raise mtDNA levels (1.796 ± 0.553 ng/ml) vs controls. However, the addition of severe trauma to mild/moderate shock (ISS > 25, BE −2.1 to −10, *n* = 32) caused a significant rise in mtDNA compared to controls but not to isolated severe trauma: 5.425 ± 1.422 ng/ml, † denotes *p* < 0.01, ANOVA/Dunnett’s. The combination of severe trauma and severe shock (ISS > 25, BE <−10, *n* = 11) resulted in the greatest rise in plasma mtDNA levels: 16.93 ± 5.894 ng/ml, § denotes *p* < 0.0001 vs controls, *p* < 0.05 vs isolated severe trauma, and *p* < 0.05 vs severe trauma and mild/moderate shock, *t*-tests. Bar graphs indicate mean with SEM.

The human cohort comprised Controls (*n* = 16), No MODS (*n* = 85) and MODS (*n* = 27), and 11 patients who died within 48 h. The demographics are reported in Table S1 in Supplementary Material. MODS patients had significantly higher ISS and BD, reflecting a higher injury burden and a higher associated mortality (no MODS 0% vs MODS 22%, *p* < 0.05). Acute lung injury was the dominant organ dysfunction component (93% of all cases of MODS at 48 h). Secondary adverse outcomes such as the development of infection, the length of stay, and mortality were also higher in the MODS group. Patients who developed MODS had significantly higher concentrations of mtDNA in their blood compared to patients who did not develop MODS (Figure 1A). Control patients had very low concentrations of circulating mtDNA. Isolated tissue injury, without shock, led to a dose-dependent increase in mtDNA release (Figure 1B). The combination of severe shock and severe trauma resulted in a substantial rise in circulating mtDNA. These data suggest that mtDNA release into the circulation occurs as a result of mechanical tissue injury and cellular injury, related to hypoperfusion. The presence of MODS was also associated with a rise in plasma IL-6 concentration (Table S1 in Supplementary Material). The influence of bacterial components as a MODS stimulus was excluded using PCR analysis of bacterial 16S rRNA in patient plasma, which demonstrated negligible concentrations (data not shown).

### Animal Study

**Figure 2:**
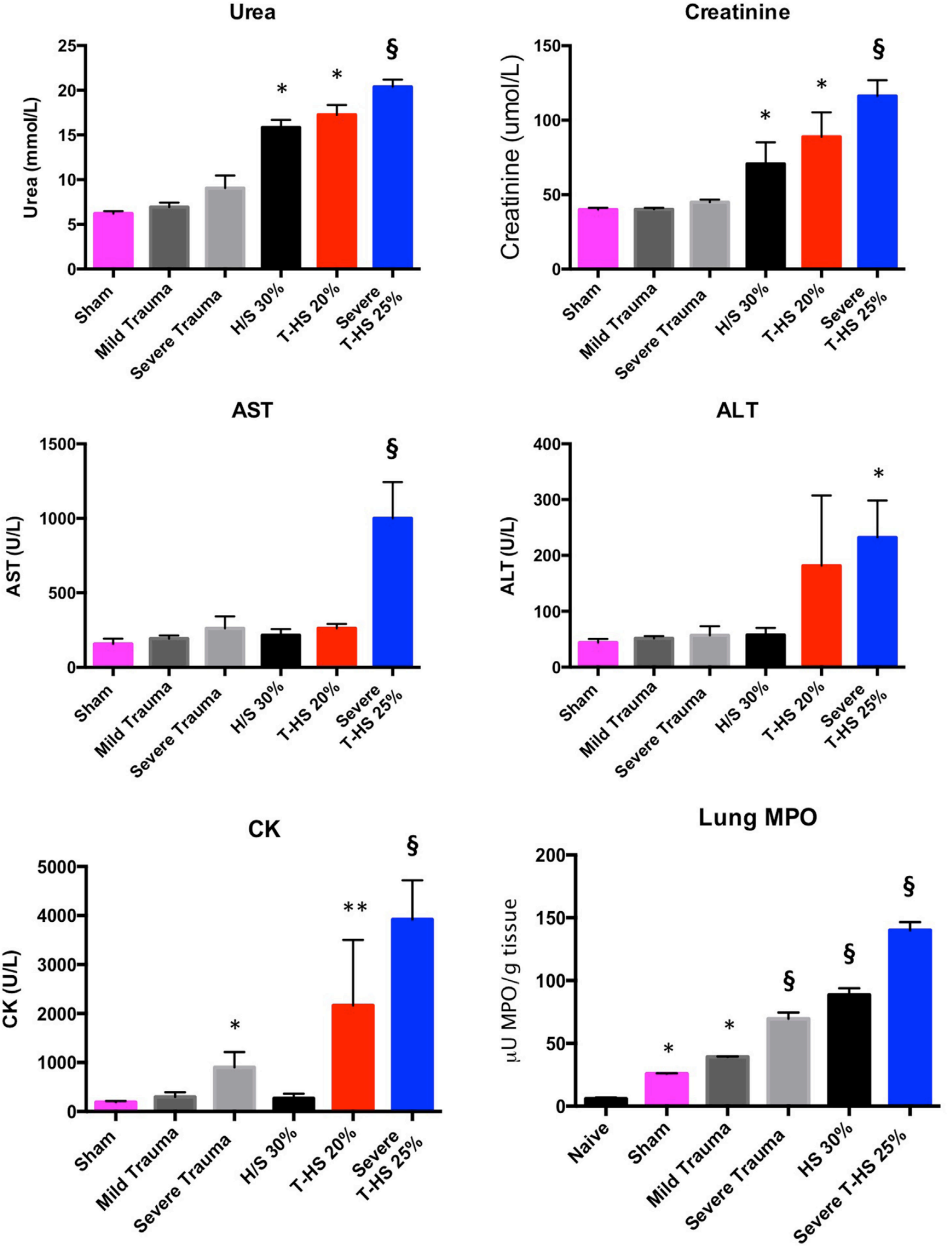
Organ injury plasma biomarkers in various models of (trauma) hemorrhage. Trauma was inflicted during −5 to 0 min. Naïve group, uninstrumented animals, *n* = 4. Sham group, instrumented animals, *n* = 8. Mild trauma group: Left leg fracture only, *n* = 6. Severe trauma: Bilateral leg fractures, 4-cm laparotomy, 10-s bilateral leg muscle crush injury, *n* = 8. HS 30% group: Bleeding of 30% circulating volume for 20 min, *n* = 8. T-HS 20% group: Bilateral leg fractures, 4-cm laparotomy, bleeding 20% circulating volume for 20 min, *n* = 8. Severe T-HS 25% group: Bilateral leg fractures, 4-cm laparotomy, 10-s bilateral leg muscle crush injury, bleeding 25% circulating volume for 35 min, *n* = 8. * denotes *p* < 0.05 vs sham; ** denotes *p* < 0.01 vs sham; § denotes *p* < 0.0001 vs sham, all *t*-tests. For lung Myeloperoxidase (MPO) symbols denote significance vs naïve animals, *n* = 4 (uninstrumented controls). For creatine kinase (CK), § also denotes *p* < 0.0001 for severe T-HS 25% vs severe trauma alone. Mean values ± SEM bars shown.

Rodents were subjected to increasing degrees of traumatic injury and shock. As can be seen in Figure 2, the development of shock resulted in varying degrees of organ injury. The addition of traumatic injury to hemorrhagic shock resulted in a more severe organ injury. Traumatic injury by itself did not result in the development of severe organ injury in these models and was associated with 100% experimental survival. Lung MPO was the most sensitive marker of increasing traumatic injury and/or shock, followed by urea/creatinine and then derangement of liver function tests (Spearman correlation analysis, data not shown). This correlates with the progression of organ dysfunction found in clinical postinjury MODS described in the literature (20) as well as in our clinical series of 139 patients.

#### Plasma DNA Levels vs Degree of Trauma and Shock

**Figure 3:**
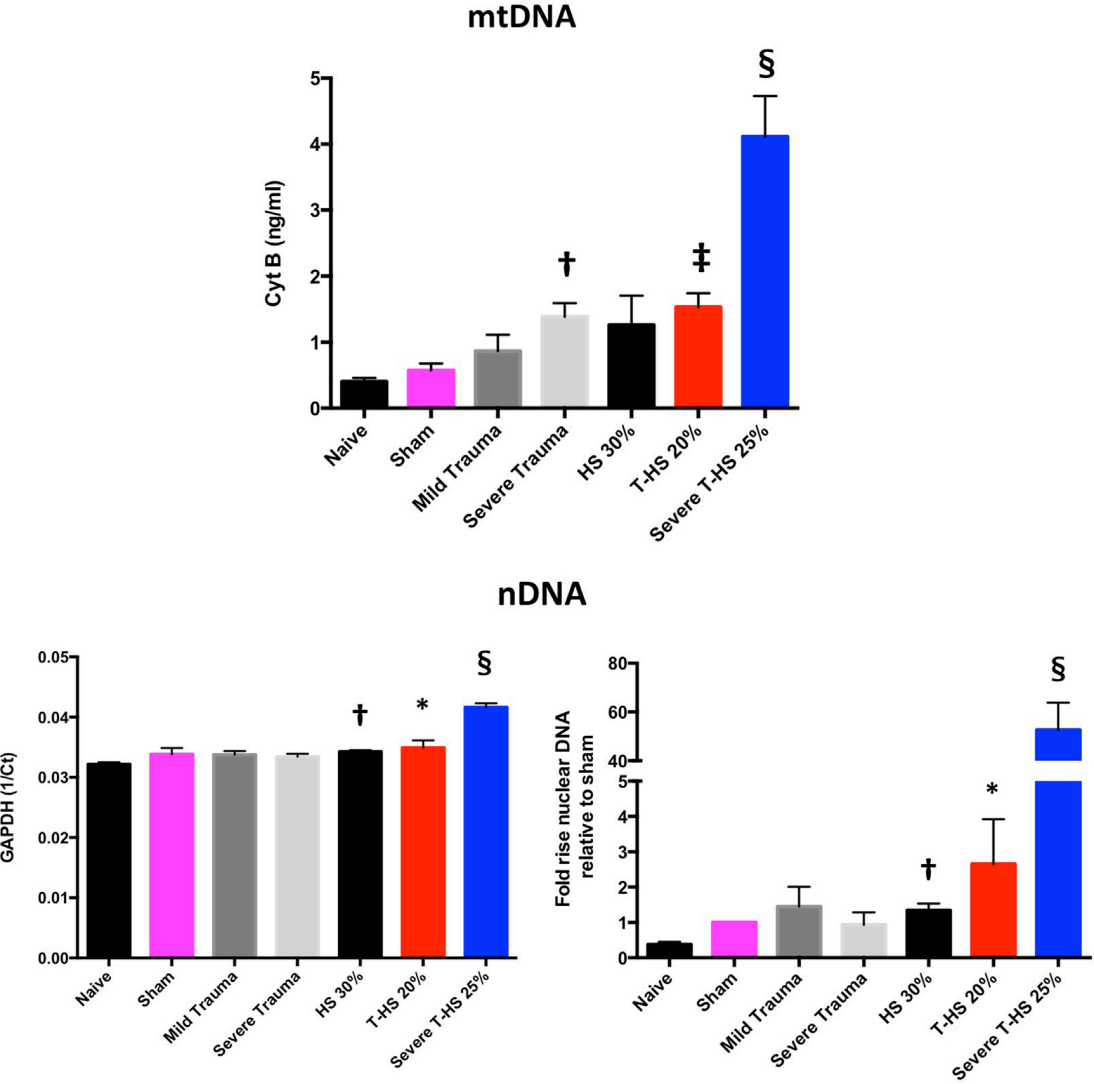
Plasma mitochondrial DNA (mtDNA) and nDNA concentrations taken at 6 h from rodents subjected to increasing degrees of traumatic injury and shock. Top panel, fully quantified mtDNA concentrations from real-time polymerase chain reaction (Cyt B). Bottom panels, nDNA, 1/Ct (GAPDH) and fold increases relative to sham concentrations displayed. There was a dose-dependent increase in mtDNA with increasing trauma severity as opposed to pure HS; † denotes *p* < 0.01 for severe trauma vs naïve animals. With increasing severity of concomitant shock there again was a dose-dependent increase in mtDNA, ‡ denotes *p* < 0.001 and § denotes *p* < 0.0001 for T-HS 20%, and severe T-HS 25%, respectively, vs naïve animals. There was moderate correlation between changes in mtDNA and nDNA concentration overall (*p* < 0.01) but important differences emerged. There was no rise in nDNA with increasing traumatic injury but pure HS 30% induced a small but significant rise in nDNA, † denotes *p* < 0.01 vs naive animals. Severe T-HS resulted in a large rise in nDNA, § denotes *p* < 0.0001 vs naïve animals. *t*-tests used. Mean values with SEM bars shown.

As can be seen in Figure 3, both mtDNA and nDNA concentrations broadly rose with increasing severity of injury. However, important differences were evident. mtDNA levels exhibited a dose-dependent increase with increasing isolated traumatic injury, whereas nDNA did not. Plasma nDNA concentrations were increased significantly with 30% hemorrhagic shock, whereas mtDNA concentrations were not. With severe combined trauma and shock both concentrations were significantly raised with respect to sham levels: 10- and 50-fold for mtDNA and nDNA, respectively. Interestingly, the 0- to 10-ng/ml range for mtDNA concentration found in this study correlates well with other studies of clinical trauma (21–23). Similar magnitude increases in nDNA levels have also been reported by others (24).

A further large correlation analysis including all measured parameters of inflammation, organ dysfunction and DNA concentrations was then carried out (Table S2 in Supplementary Material). MtDNA and nDNA were only moderately correlated to each other (*p* < 0.01), which lead to some divergent findings in this analysis. Interestingly, of all the variables measured (except lung IL-6), mtDNA was the most highly correlated to lung MPO levels (*p* < 0.001). NDNA, by contrast, was weakly correlated with lung MPO (*p* = 0.051). MtDNA was highly correlated with urea concentrations (*p* < 0.0001) and moderately correlated with plasma IL-6 and the other organ function scores (*p* <0.05 to <0.01). NDNA, on the other hand, was highly correlated with plasma IL-6 and the non-lung organ injury markers (*p* < 0.0001). Although this does not confirm causation, it supports a large body of work suggesting that lung injury is particularly driven by mtDNA concentrations rather than nDNA (8, 23, 25–30). By contrast, these studies have shown nDNA to be immunologically inert. The high expression of CpG repeats present in mtDNA and relative CpG suppression in nDNA also adds weight to this view. There is some doubt, however, as to the biological plausibility of injections of pure mtDNA or nDNA as a relevant traumatic insult. Although tempting to conclude that circulating nDNA in trauma is inert, this view downplays a significant body of work that suggests nDNA is inflammogenic in certain circumstances, most evident in studies of chronic autoimmune diseases (31–33). This is particularly evident when the nDNA is fragmented (as would be the case as a product of cellular necrosis), when it is present in a double-stranded form and when it is associated with histones in the form of nucleosomes (34). MtDNA probably has a greater inflammatory potential with less purity (35), when derived as a synthetic PCR product (25, 27) when co-present with other mitochondrial molecules such as TFAM or formyl peptides (36, 37), such as when derived from whole mitochondria (8) or when administered in high concentrations (38). These factors are not readily simulated in experimental conditions. In summary, these results suggest that the mechanisms of release of nDNA and mtDNA are different in trauma hemorrhage. Clinically, elevated nDNA levels may simply be reflective of general illness severity, hypoperfusion status or be a marker of the release of other DAMPs (24), whereas mtDNA release appears to be more closely related to cell disruption and necrosis, at least in the early postinjury phase.

#### Time Course of Plasma DNA Levels in Trauma Hemorrhage

The time course of plasma DNA levels appears to depend on the degree of trauma and/or shock sustained. Isolated trauma generates a disproportionate rise in mtDNA within 2 h post injury [unpublished data from our group, also reported by Ref. (21, 39)].

The peak of mtDNA post-combined trauma hemorrhage clinically has been found to occur within the first 24 h of injury and levels remained elevated for a week in several studies (24, 39–41). This concurs with an animal study which showed that resuscitated trauma hemorrhage resulted in a peak of mtDNA at around 24 h as well (42). Delayed rises in mtDNA at 3–5 days post injury have also been noted and attributed to NETosis (43, 44).

#### Circulating Nucleic Acid Concentration Is Decreased With NASP Treatment in T-HS

**Figure 4:**
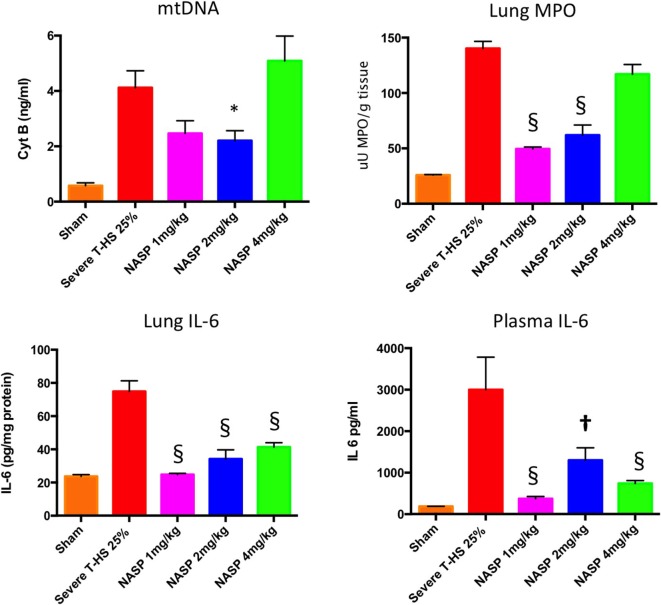
A 6-h plasma mitochondrial DNA (mtDNA), lung inflammation scores and systemic IL-6. Cell-free mtDNA was measured with real time polymerase chain reaction using cytochrome B as the target gene: Severe T-HS 25% resulted in an approximately eightfold increase in mtDNA compared to sham, 0.57 (±0.1) to 4.1 (±0.6) ng/ml, *p* < 0.0001, *t*-test. The addition of nucleic acid scavenging polymer (NASP) (hexadimethrine bromide) 1 mg/kg post injury resulted in a non-significant trend toward reduced mtDNA, mean 2.46 (±0.46) ng/ml, *p* = 0.06. NASP 2 mg/kg reduced circulating plasma mtDNA by half to 2.2 (±0.36) ng/ml, * denotes *p* < 0.05. NASP 4 mg/kg resulted in an increased mean mtDNA 5.09 (±0.9) ng/ml relative to untreated T-HS 25%, *p* > 0.05. Lung MPO: untreated T-HS 25% showed a marked increase in lung MPO compared to shams, 140 (±0.5) vs 25.8 (±0.8) uU MPO/g, *p* < 0.0001. There was significant attenuation of lung MPO in the NASP 1 and 2 mg/kg groups, 49.4 (±0.4) and 61.9 (±0.9) uU MPO/g respectively, § denotes *p* < 0.0001, but not in the NASP 4 mg/kg group. Lung IL-6 concentration was measured by ELISA: untreated T-HS 25% resulted in a significant increase in Lung IL-6 from sham levels, 74.9 (±6.5) vs 23.7 (±1.1) pg/mg protein, *p* < 0.0001. There was significant attenuation in lung IL-6 with all three doses used, 24.7 (±0.76), 34.2 (±5.5), and 41.3 (±2.3) pg/mg protein, respectively, § denotes *p* < 0.0001 vs untreated T-HS 25%. Systemic IL-6 concentration was measured by ELISA: There was a marked increase in plasma IL-6 in untreated T-HS compared to controls, 2,999 (±782) pg/ml vs 186 (±2) pg/ml, *p* < 0.0001. There was significant attenuation of IL-6 release with all three doses used: 373 (±51), 1,299 (±298), and 738 (±69) pg/ml, respectively, † denotes *p* < 0.01 and § denotes *p* < 0.0001 vs untreated T-HS 25%, *t*-tests throughout. Mean values with SEM bars shown.

**Figure 5:**
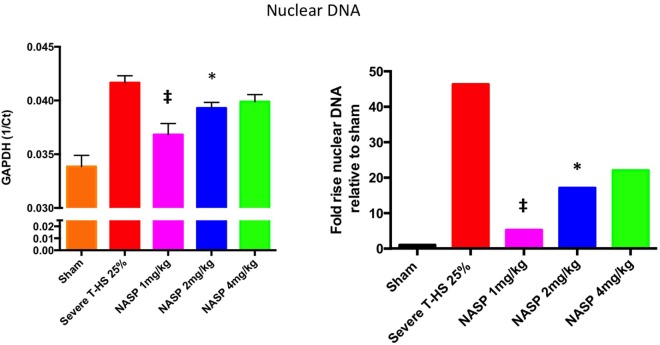
A 6-h plasma nuclear DNA concentrations. Cell-free plasma nDNA was measured by real time polymerase chain reaction using GAPDH as the target gene. *Y*-axis is denoted as the reciprocal of the threshold count, Ct, the PCR cycle at which the rate of PCR product starts to rise exponentially, which corresponds to the amount of starting template. The right hand graph illustrates the corresponding fold increase in nDNA relative to sham. There was a 45-fold increase in nDNA concentration relative to sham, *p* < 0.0001. Nucleic acid scavenging polymer (NASP) (hexadimethrine bromide) 1 mg/kg resulted in significant attenuation of this rise to fivefold sham levels; ‡ denotes *p* < 0.001. NASP 2 mg/kg resulted in lesser but significant attenuation to 17-fold sham levels, * denotes *p* < 0.05. Attenuation with NASP 4 mg/kg was not significant statistically. *t*-tests used. Mean values with SEM bars shown.

As can be seen in Figure 4, circulating plasma mtDNA at 6 h increased 10-fold in untreated trauma hemorrhage compared to sham controls. The use of NASP 1 mg/kg post injury resulted in a non-significant reduction in mtDNA (*p* = 0.06); NASP 2 mg/kg caused a significant reduction in mtDNA by about 50% (*p* = 0.015). Interestingly, NASP 4 mg/kg showed an approximately unchanged mtDNA concentration compared to controls. Figure 5 illustrates the corresponding changes in nDNA. Untreated severe trauma hemorrhage resulted in a 50-fold rise in nDNA. NASP 1 mg/kg resulted in attenuation in nDNA levels to approximately 5-fold sham levels, followed by a 17- and 22-fold rise for NASP 2 and NASP 4 mg/kg groups, respectively. Lung MPO appears to be a very sensitive marker of lung injury that is responsive to higher doses of NASP *per se*, probably reflecting toxicity and cell necrosis at 4 mg/kg dosing (Figure 10). mtDNA release could be a result of this toxicity itself reflecting cellular oxidative stress and damage. Similar toxicity has been noted with other cationic agents (29, 45, 46). Bacterial contamination was excluded in all studies with the use of PCR targeting bacterial 16S rRNA (Figure S3 in Supplementary Material).

#### NASP Treatment Produces Broad Anti-Inflammatory Action at the Transcriptional Level in T-HS

**Figure 6:**
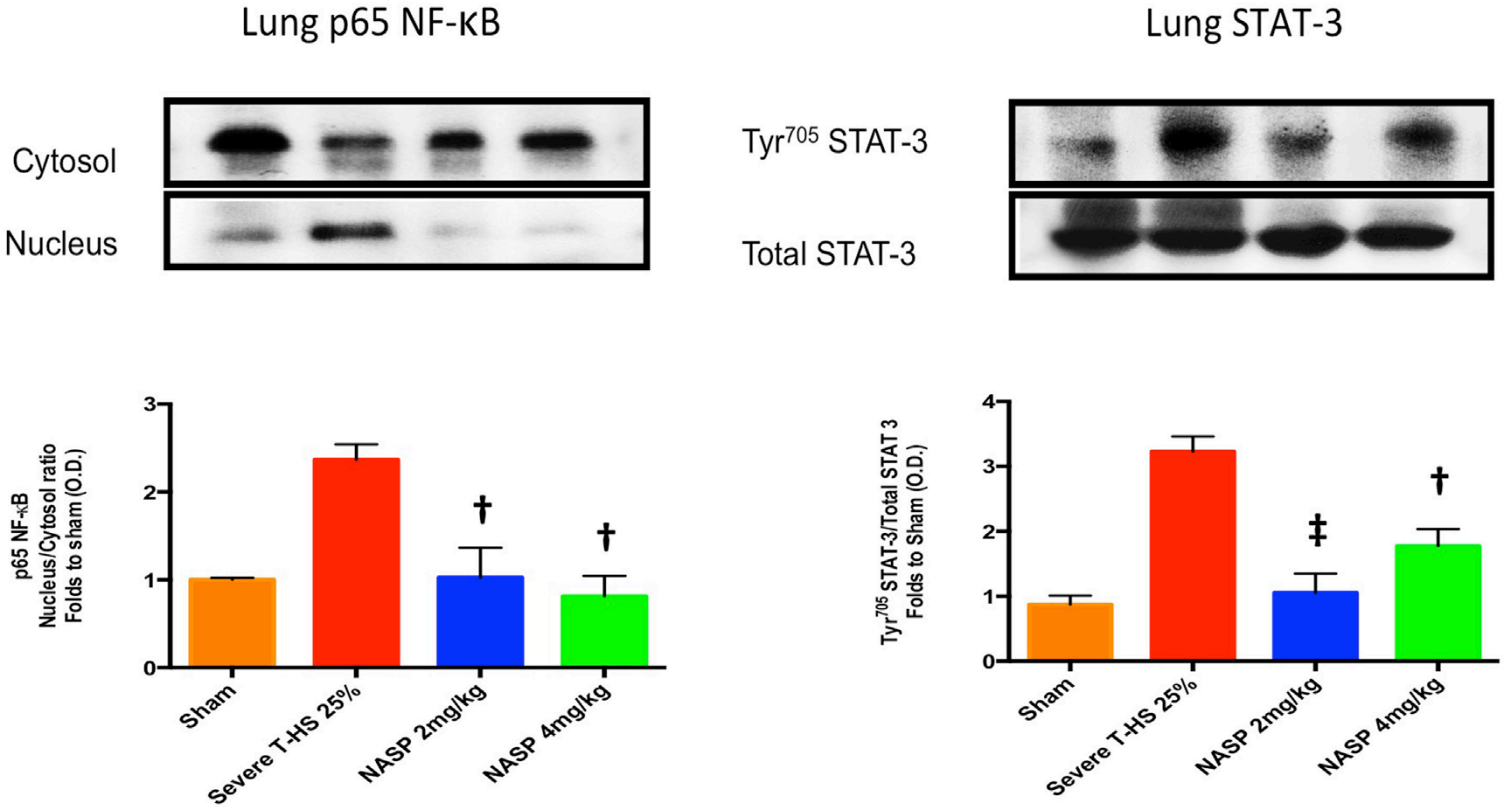
Western blot analysis of lung homogenates. Phosphorylated NF-κB levels were markedly increased at 6 h post T-HS with attenuation to sham levels with nucleic acid scavenging polymer (NASP) (hexadimethrine bromide) 2 and 4 mg/kg dosing, † denotes *p* < 0.01 vs untreated T-HS 25%. Phosphorylated STAT-3 levels were similarly attenuated by NASP 2 mg/kg, ‡ denotes *p* < 0.001 vs untreated T-HS 25%, less so with NASP 4 mg/kg, † denotes *p* < 0.01 vs untreated T-HS 25%. *n* = 3–4 animals per group. ANOVA/Tukey’s. Bar graphs indicate mean values with SEM. NASP 1 mg/kg data not available.

IL-6 was the most consistently elevated cytokine measured in this study. Of note, other cytokines and DAMPs were measured at 6 h in this experiment including IL-1β, TNF-α, HMGB1, and TFAM but extremely low or undetectable levels were found in all groups (data not shown). IL-6 exerts its action *via* the signal transducer gp130 leading to the activation of the STAT and MAPK cascades (47). TLR-9 is known to signal *via* Myd88 which then activates NF-κB signaling to produce a broad inflammatory phenotype (29). NF-κB is also a strong inducer of IL-6 (48). Western blot analysis of lung homogenates for phosphorylation of NF-κB and STAT-3 was performed (Figure 6). There was a marked attenuation of the phosphorylation of NF-κB to sham levels with both NASP 2 mg/kg and NASP 4 mg/kg. With regard to STAT-3, NASP 2 mg/kg produced an attenuation of the phosphorylation to sham levels; NASP 4 mg/kg produced less but still significant attenuation. No loss of anti-inflammatory action was noted at the higher dose of NASP used.

#### NASP Treatment Improves Lung Histological Appearance in T-HS

Trauma hemorrhagic shock leads to cellular stress, failure of mitophagy, and autophagy, increasing degrees of oxidative stress, translocation of mtDNA to the cytosol, and activation of apoptotic cell death pathways (49, 50). When the ischemia is prolonged and/or reperfusion injury supervenes, cellular necrosis occurs with resultant movement of mtDNA into the extracellular space. Apoptosis is known to be associated with the release of oxidized mtDNA into the cytosol, where it binds to the NLRP3 inflammasome causing local and systemic inflammation (51).

**Figure 7:**
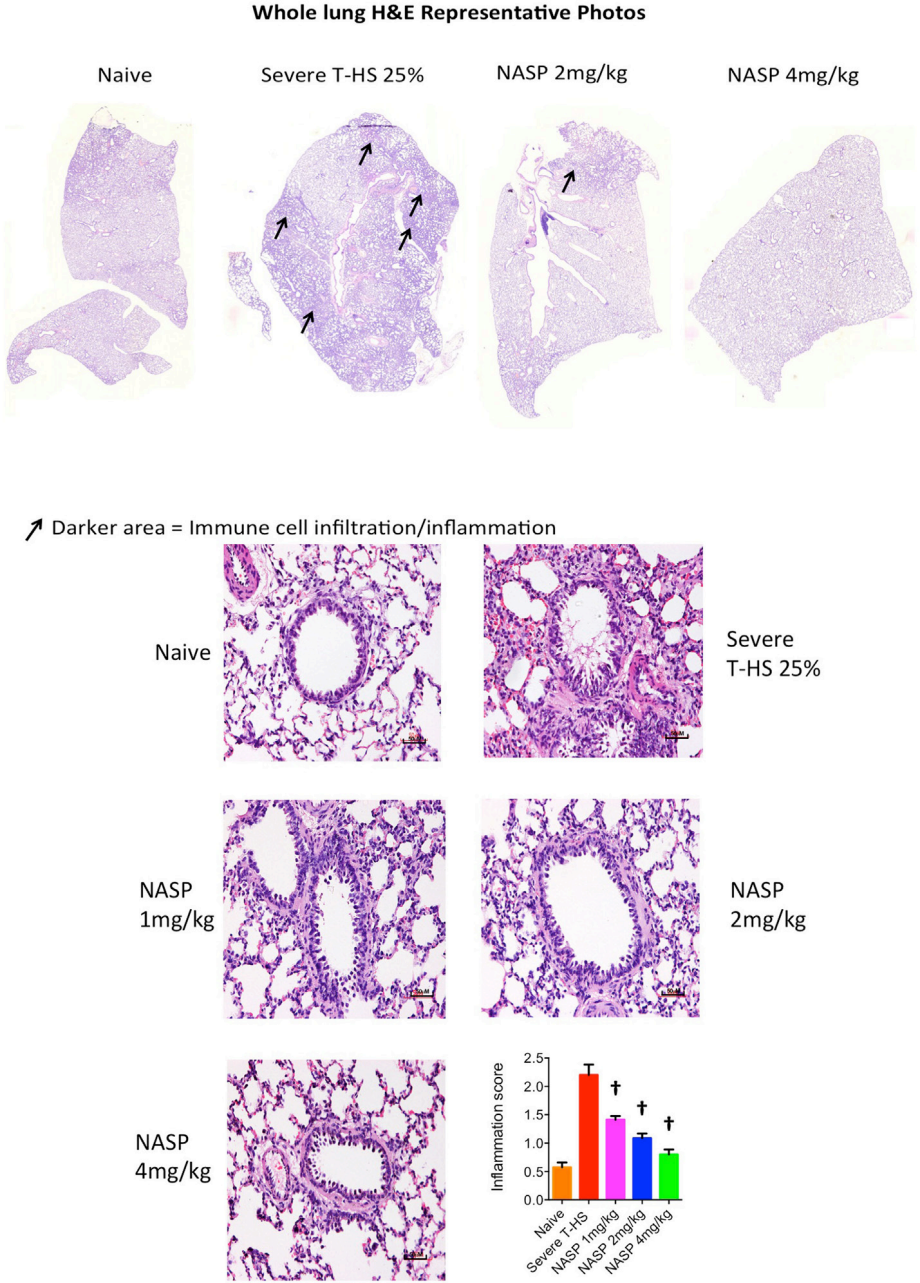
Nucleic acid scavenging polymer (NASP) (hexadimethrine bromide) treatment of rodent T-HS improved lung histological appearances. Hematoxylin & eosin (H&E) staining to measure cell infiltration into the airway, an indicator of airway inflammation. NASP treatment showed a dose-dependent attenuation of cellular infiltration. † denotes *p* < 0.01 vs untreated severe T-HS 25%, *t*-test. *n* = 4–7 animals per group. Mean values with SEM bars shown. Scale bars represent 50 µm.

**Figure 8:**
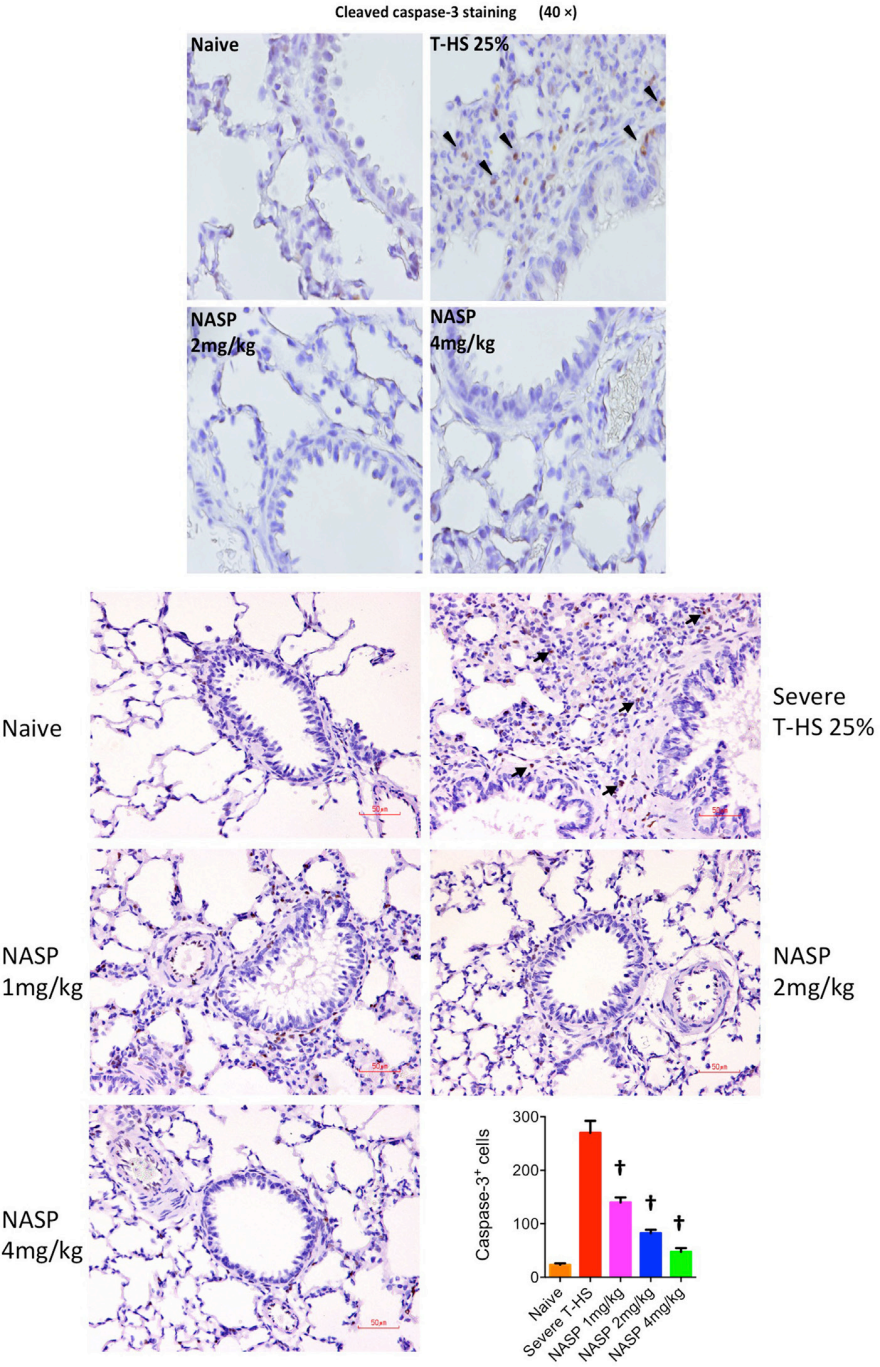
Nucleic acid scavenging polymer (NASP) (hexadimethrine bromide) treatment of rodent T-HS improved lung histological appearances. Cleaved caspase-3 staining was used to evaluate apoptotic cell death. NASP treatment showed a dose-dependent reduction of apoptotic cell death. † denotes *p* < 0.01 vs untreated severe T-HS 25%, *t*-test. *n* = 4–8 animals per group. Dark arrows indicate cleaved caspase-3 cells. Scale bar represents 50 µm.

**Figure 9:**
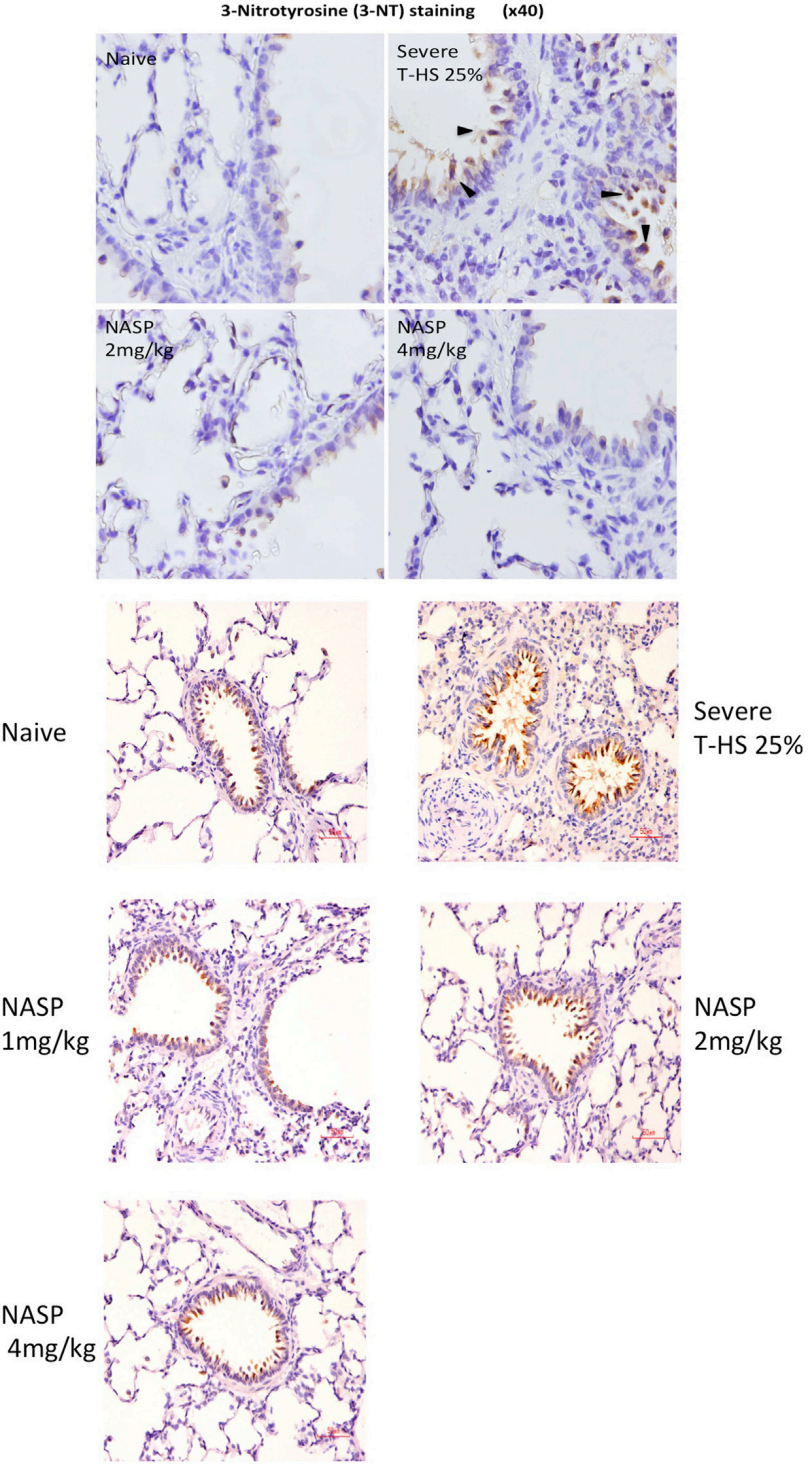
Nucleic acid scavenging polymer (NASP) (hexadimethrine bromide) treatment of rodent T-HS improved lung histological appearances. Staining for 3-nitrotyrosine (3-NT), a marker of peroxynitrite production and hence oxidative stress, was used to evaluate oxidative injury. There was a dose-dependent improvement in lung histological appearance with increasing doses of NASP (not quantified). Black arrows indicate 3-NT-positive stained cell. Red scale bar represents 50 µm.

Immune cell infiltration, apoptotic cell death, and oxidative injury in lung tissue were assessed by the use of H&E, cleaved caspase-3, and 3-Nitrotyrosine (3-NT) staining, respectively (Figures 7–9). There was a broad protective effect evident with all three stains with NASP use and evidence of a dose-dependent protective effect. Again, no loss of protection was noted at the highest dose of NASP used.

#### NASP Toxicity Study

**Figure 10:**
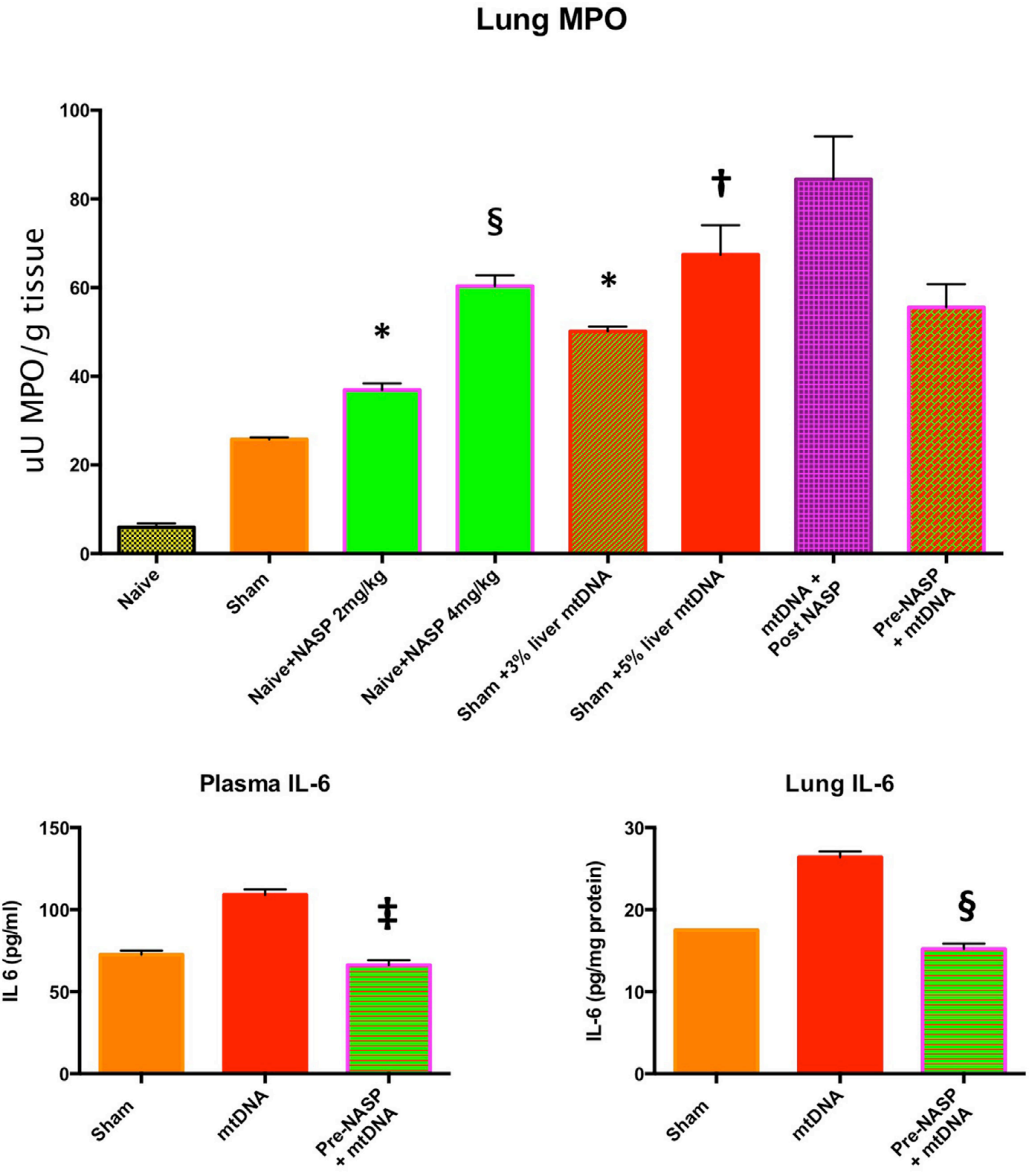
Lung myeloperoxidase (MPO), lung and plasma IL-6. Lung MPO (Top): Injection of nucleic acid scavenging polymer (NASP) (hexadimethrine bromide) 2 and 4 mg/kg into naïve rats. Lungs sampled at 6 h. There were significant increases in MPO with both doses compared to naïve animals indicating lung toxicity at the higher dose especially, * denotes *p* < 0.05, § denotes *p* < 0.0001. Pure mitochondrial DNA (mtDNA) extracted from 3 and 5% rat liver by total weight was injected into sham animals with dose-dependent increases in lung MPO compared to sham animals, * denotes *p* < 0.05, † denotes *p* < 0.01. Then, pure mtDNA extracted from 5% liver was injected into shams followed by NASP 2 mg/kg 30 min later; there was no significant change in MPO found. Finally, shams were injected with NASP 2 mg/kg followed by 5% liver pure mtDNA injection 15 min later. No significant alteration in lung MPO was found. *t*-tests throughout. *n* = 3–5 animals per group. Plasma and lung IL-6 (Bottom) were both measured by ELISA: Injection of pure mtDNA extracted from 5% liver resulted in a significant rise in both plasma and lung IL-6 concentrations, *p* < 0.01. Pretreatment of sham animals with NASP 2 mg/kg followed by injection of pure mtDNA extracted from 5% liver 15 min later resulted in the attenuation of both values to sham levels, ‡ denotes *p* < 0.001, § denotes *p* < 0.0001 vs pure mtDNA, *t*-tests. *n* = 3–5 animals per group. Overall, MPO appears to be a very sensitive marker of lung injury. Pretreatment with NASP attenuated other inflammatory marker increases with pure mtDNA injections. Mean values with SEM shown.

*De novo* toxicity was evaluated by the injection of NASP 2 and NASP 4 mg/kg into healthy animals (Figure 10). Significant increases in lung MPO were noted with increasing doses of NASP compared to naïve animals. This is in keeping with the known and predicted side effects of this agent given its charge chemistry and likely affinity for binding to pulmonary vascular endothelium. There was no evidence of significantly increased renal, liver or muscle injury with the higher NASP 4 mg/kg dose (*p* > 0.10, data not shown). The kidney has been noted to be particularly resistant to the inflammatory potential of mtDNA challenge in a previous study in mice and rats (27). There was a trend toward increased mtDNA concentrations with NASP 4 mg/kg (*p* = 0.06) and no significant increase in nDNA concentrations (Figure S2 in Supplementary Material). Overall, this suggests possible cytotoxicity with this dose of NASP, which could potentially lead to a feedforward cycle of mtDNA-propagated inflammation.

#### mtDNA Challenge in Healthy Animals

Pure mtDNA extracted from rodent liver was injected into healthy animals and plasma and organs were sampled at 6 h (Figure 10). Two doses of mtDNA were chosen, 3 and 5% of liver by weight, because they had been used extensively by Hauser’s group and produced clinical range plasma mtDNA concentrations with subsequent organ injury (8, 42). The pure mtDNA extracts were proven to be infection-free with the use of PCR for bacterial 16S rRNA (data not shown). A dose-dependent increase in lung MPO was detected with an increasing dosage of mtDNA compared to shams. However, post treatment with NASP 2 mg/kg 30 min after mtDNA challenge failed to attenuate this rise in MPO. Similarly, pretreatment with NASP 2 mg/kg 15 min before mtDNA challenge failed to attenuate the lung injury as measured by MPO. The reasons for the inability to rescue MPO induced lung injury are not clear but include extreme sensitivity of the lung MPO test to measure inflammation, crude modeling of the clinical injury with a single bolus of mtDNA, and susceptibility of the animal lung to the toxicity of the treatment itself.

A small but significant rise in plasma IL-6 and lung IL-6 was evident with the 5% liver mtDNA dose (Figure 10). Other plasma organ injury markers were not elevated. However, pretreatment with NASP 2 mg/kg attenuated the rise in lung and plasma IL-6 to sham levels.

**Figure 11:**
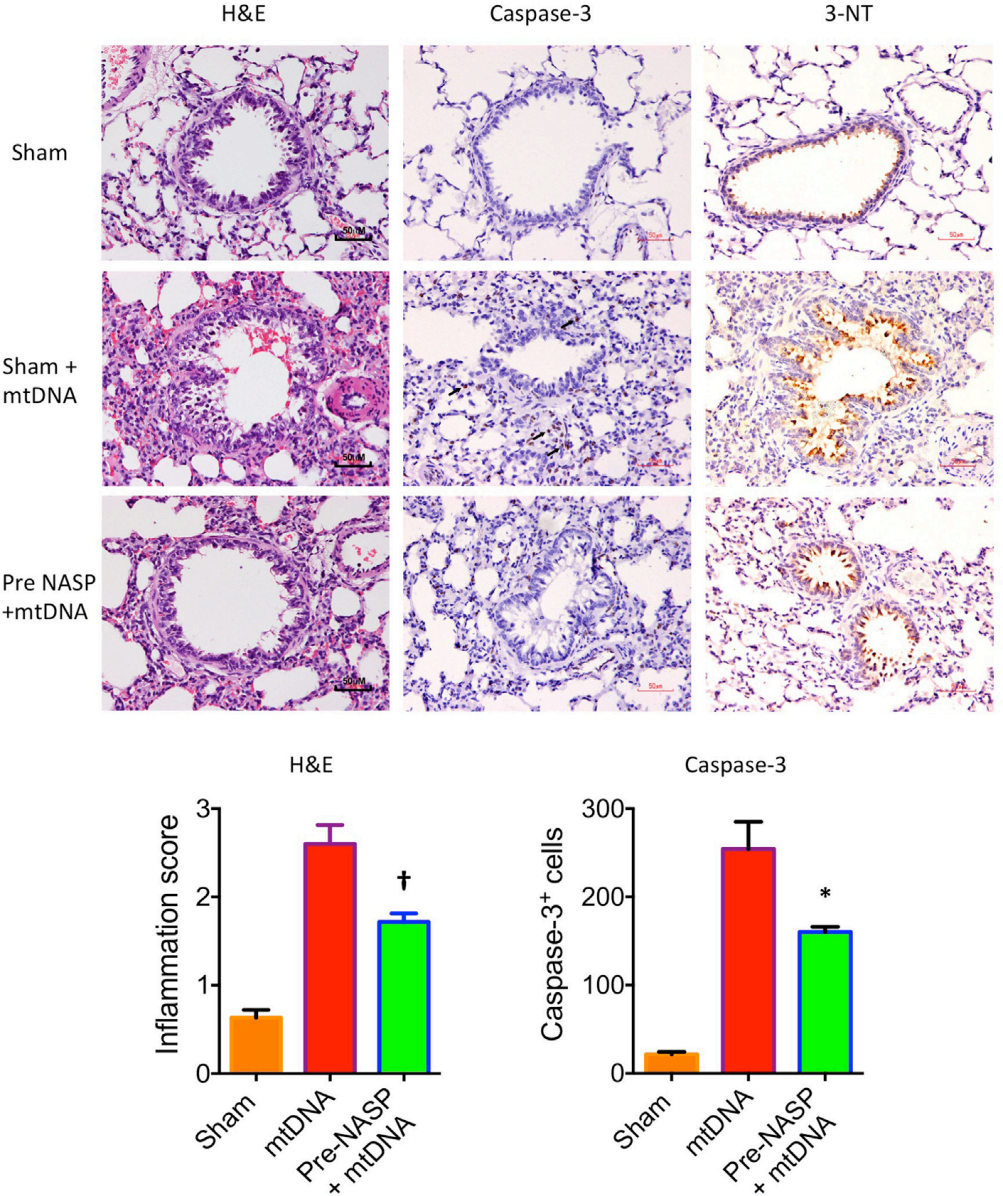
Nucleic acid scavenging polymer (NASP) (hexadimethrine bromide) 2 mg/kg pretreatment followed by 5% liver pure mitochondrial DNA (mtDNA) injections into sham animals attenuated lung injury on histological examination with hematoxylin & eosin (H&E), caspase-3M and 3-Nitrotyrosine (3-NT) staining. * denotes *p* < 0.05, † denotes *p* < 0.01, both vs pure mtDNA injection alone, *t*-tests. *n* = 3–5 animals per group. Mean values with SEM bars shown.

Further histological examination of lungs from this experiment using H&E, cleaved caspase-3 and 3-NT stains further confirmed that the injection of pure mtDNA into healthy animals caused moderately severe acute lung injury on histological appearances. NASP pretreatment was able to attenuate inflammatory cellular infiltration, apoptotic cell death and oxidative cellular injury in lung tissue in a dose-dependent manner (Figure 11).

#### Other Plasma Organ Injury Markers

**Figure 12:**
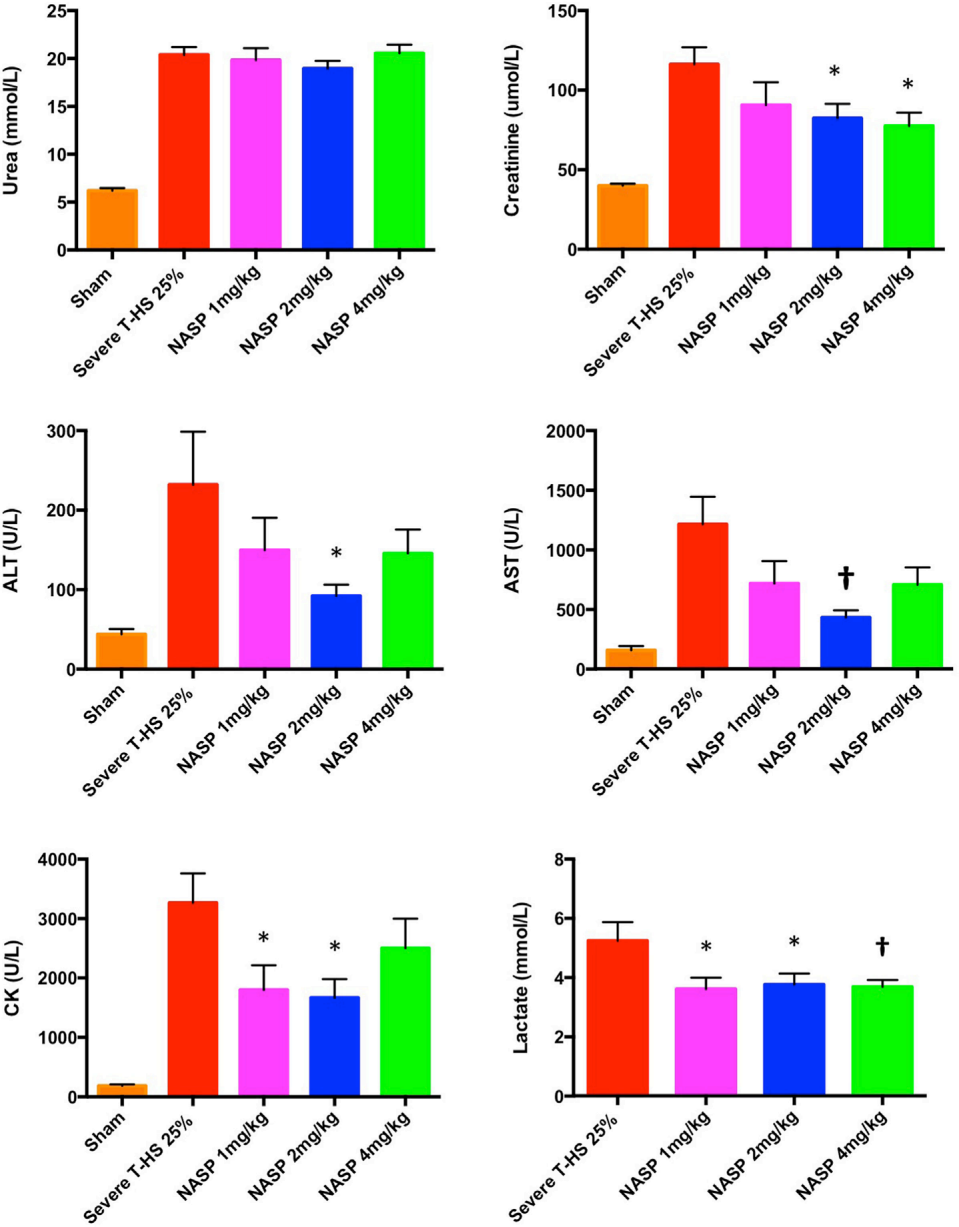
Other organ injury markers at 6 h. Urea: there was no detectable difference in urea concentration with an increasing dose of nucleic acid scavenging polymer (NASP) (hexadimethrine bromide) used in severe T-HS 25%. Creatinine: renal protection was evident with NASP 2 and 4 mg/kg doses. Alanine aminotransferase (ALT) and aspartate aminotransferase (AST): there was a significant attenuation of liver injury evident with NASP 2 mg/kg dosing. Creatine kinase (CK): there was a significant attenuation of muscle injury with both NASP 1 and 2 mg/kg doses. Lactate: there was a significant reduction in 6 h lactate with all three doses NASP used. * denotes *p* < 0.05, † denotes *p* < 0.01 was untreated severe T-HS 25%, *t*-tests. *n* = 12–18 animals per group. Mean values with SEM bars shown. Overall, the NASP 2 mg/kg group produced the most consistent multiple organ protection in severe T-HS 25%.

The results of this array of tests were more mixed (Figure 12). All three doses produced a similarly statistically reduced 6-h lactate compared to untreated T-HS animals. As the bleeding phase, injury phase and post mortem examinations (to exclude large limb hematomas or i.p. bleeding, for example) were similar in all groups, this improvement in lactate supports a broad cellular protective mechanism with NASP treatment. However, multiple organ protection was most consistently shown with NASP 2 mg/kg.

## Discussion

We have shown that the release of mtDNA is sufficient for the development of severe organ injury. HDMBr scavenging of circulating mtDNA (and nDNA) in an *in vivo* model of trauma hemorrhage is associated with protection from severe organ injury. This suggests that HDMBr could also rescue patients from trauma-induced MODS.

Hexadimethrine bromide has previously been safely and extensively used as an antiheparin agent in cardiopulmonary bypass surgery in doses up to 5 mg/kg (52–61). Therefore, a safety profile exists clinically for HDMBr which could allow a feasible translation of its use as a NASP in trauma hemorrhage.

Overall, the results pertaining to acute lung injury and the use of NASP in T-HS suggest that HDMBr has definite lung protective properties, most convincingly at 1 and 2 mg/kg doses. Higher doses appear to elicit toxicity as evidenced by increased lung MPO concentrations and possibly higher plasma mtDNA levels. Polycationic compounds in general have been shown to accumulate in lung tissue in particular (29). However, this toxicity is neither demonstrated in the cytokine profile nor on histological examination of lungs from rats subjected to trauma hemorrhage and treated with HDMBr. The expected toxicity of cationic agents such as HDMBr would include plasma membrane destabilization and apoptotic cell death signaling. These features have not been demonstrated in this study in the context of rescue from severe trauma hemorrhage induced injury. Clearly, there is a risk–benefit profile and therapeutic index to be established in further studies. The mechanistic mtDNA challenge experiments in HDMBr-pretreated rodents confirm that mtDNA is an important mediator in the pathogenesis of trauma hemorrhage induced lung injury and that mtDNA can be targeted by a NASP such as that used here. However, circulating nDNA is also reduced in these experiments, so an effect from nDNA cannot be fully excluded.

The ability of HDMBr to bind free plasma mtDNA could be a large part of its protective mechanism. This is also supported by recent studies in which NASPs were immobilized on microspheres or incorporated into a nanofiber mesh and co-incubated with cells and CpG DNA to produce cellular protection (62, 63).

The exact nature of HDMBr–DNA interactions and their subsequent cellular trafficking remains poorly elucidated. HDMBr is a nanoscale compound which has a well-recognized ability to condense DNA into nanocomplexes for delivery into cells (10). Its ability to disrupt plasma membranes and increase membrane permeability to itself and other molecules probably also plays a part in its cellular uptake (64). Despite being positively charged and still retaining most of this charge with the addition of cargo such as DNA, these nanomolecules are small enough to penetrate within cells. HDMBr has one of the smallest molecular weights in this class which probably aids its cellular entry, although no direct evidence exists for the internalization of HDMBr itself. These effects have been indirectly measured in the case of HDMBr–CpG interactions; CpG, in the presence of HDMBr treated cells, was seen to localize in the cytoplasm and the nucleus rather than co-localize at the endosomal compartment where TLR-9 is located (10). Other than entry *via* the (disrupted) plasma membrane, polycation–DNA complexes have been shown to be internalized *via* endocytic and phagocytic routes into vesicles where they then destabilize endosomal membranes or act as proton sponges, which then release the complexes into the cytoplasm. Lysosome perforation with lysosomal enzyme leakage, and mitochondrial permeabilization and mitochondrially mediated apoptosis with resultant cytochrome c leakage have also been demonstrated (46, 65). Recent work has documented a direct polycationic toxicity due to its direct binding with Na^+^/K^+^-ATPase and its resultant impairment. This resulted in cell necrosis with the leakage of mtDNA and canonical TLR-9/MyD88 dependent inflammation (29). A trend toward increased mtDNA concentrations was found in our NASP 4 mg/kg T-HS and toxicity experiments. Necroptosis, NETosis, and pyroptosis can also liberate mtDNA and may account for this rise (33).

Important limitations of this study should be noted. It was underpowered to detect differences in survival. The study, although randomized for the intervention, was not blinded; however, every effort was made to standardize the trauma and hemorrhage phases for each animal (Figure S1 in Supplementary Material). Inspection of laparotomy wounds showed them all to be dry at the termination of the experiments, and post mortem examinations of all animals excluded i.p. bleeding and limb hematomas related to fracture sites. However, an analysis of coagulation was not carried out; this would be useful given the unknown effects of HDMBr on the coagulation system when used in severe trauma hemorrhage. Finally, the experimental model was a short-term unresuscitated trauma hemorrhage rodent model which does not mirror the clinical course exactly. It does have a greater relevance for the military or extreme pre-hospital scenario, where extraction times are prolonged. We identified heparin as a potent inhibitor of PCR in a previous model using shed heparinized blood resuscitation [unpublished data and also reported by others (66)]. We initially felt that this would best represent modern damage-control resuscitation strategies, which includes the use of permissive hypotension, avoidance of large volumes of crystalloid or colloid, and the use of balanced ratios of blood products during massive blood transfusion. Further investigation of this compound using larger more relevant models, potentially with citrated blood resuscitation, would appear to be sensible next steps. Despite these limitations, mtDNA concentrations at 2 h post clinical injury and 6 h post injury in the animal appear to show a degree of similar predictive value for the development of MODS or severe organ dysfunction, respectively. Interestingly, Simmons et al. (40) reported on clinical trauma and found that baseline mtDNA concentrations (taken within 8 h of injury) were broadly comparable to subsequent levels taken over the first 7 days as well and were highly associated with the development of MODS and mortality.

Further exploration of HDMBr and other members of this class of therapeutic agent is required to build on the proof of concept as explored in this paper. If successful, this could potentially provide a much-needed treatment in critically injured trauma patients.

## Ethics Statement

Animal study: all experiments were carried out using male Wistar rats (Charles River, UK) weighing between 280 and 350 g. Animals received a standard diet and free access to water during a 7-day adaptation period after transport into the laboratory from the supplier. This was performed in accordance with Home Office Guidance in the Operation of the Animals (Scientific Procedures) Act 1986 and the Guiding Principles in the Care and Use of Animals published by the American Physiological Society and under the approval of Queen Mary University London. Human study: The Royal London Hospital (RLH) is a busy, urban Major Trauma Center and home to The London Air Ambulance. Trauma research is conducted at the RLH by The Barts Centre for Trauma Sciences (C4TS), Queen Mary University, London, who have been recruiting to a prospective, observational cohort study called the Activation of Coagulation and Inflammation in Trauma 2 (ACIT2) since 2008, to investigate the host response to traumatic injury. Trauma patients are recruited on admission to the emergency department if they present within 2 h of injury. Blood samples are drawn on admission, 24 (±1 h) and 72 h, and participants are seen daily until death or discharge. Written consent is obtained from all subjects although, if incapacitated, temporary consent can initially be obtained from a legally appointed representative (LAR). The study has approval from the National Health Service Research Ethics Committee (REC): 07/Q0603/29.

## Author Contributions

AA designed and conducted the basic science model and experiments, DNA extraction, performed/quantified PCR, ELISA assays, performed the clinical data analysis, and wrote the manuscript. JM analyzed the clinical data and performed clinical assays, and contributed to the manuscript. KI and CH performed the PCR on the clinical samples and advised on PCR optimization. MC and FC performed the lung tissue MPO assay, ELISAs, and Western blots. WLW, TC, and WSFW performed the lung histological and immunohistochemical analyses. CT co-supervised the project. KB co-supervised the project and co-wrote the manuscript.

## Conflict of Interest Statement

The authors declare that the research was conducted in the absence of any commercial or financial relationships that could be construed as a potential conflict of interest.
