# Supplementary figures and images for: Scavenging Circulating Mitochondrial DNA as a Potential Therapeutic Option for Multiple Organ Dysfunction in Trauma Hemorrhage
Source: Front Immunol. 2018 May 8;9:891. doi: 10.3389/fimmu.2018.00891 (PMC5951958; doi:10.3389/fimmu.2018.00891)

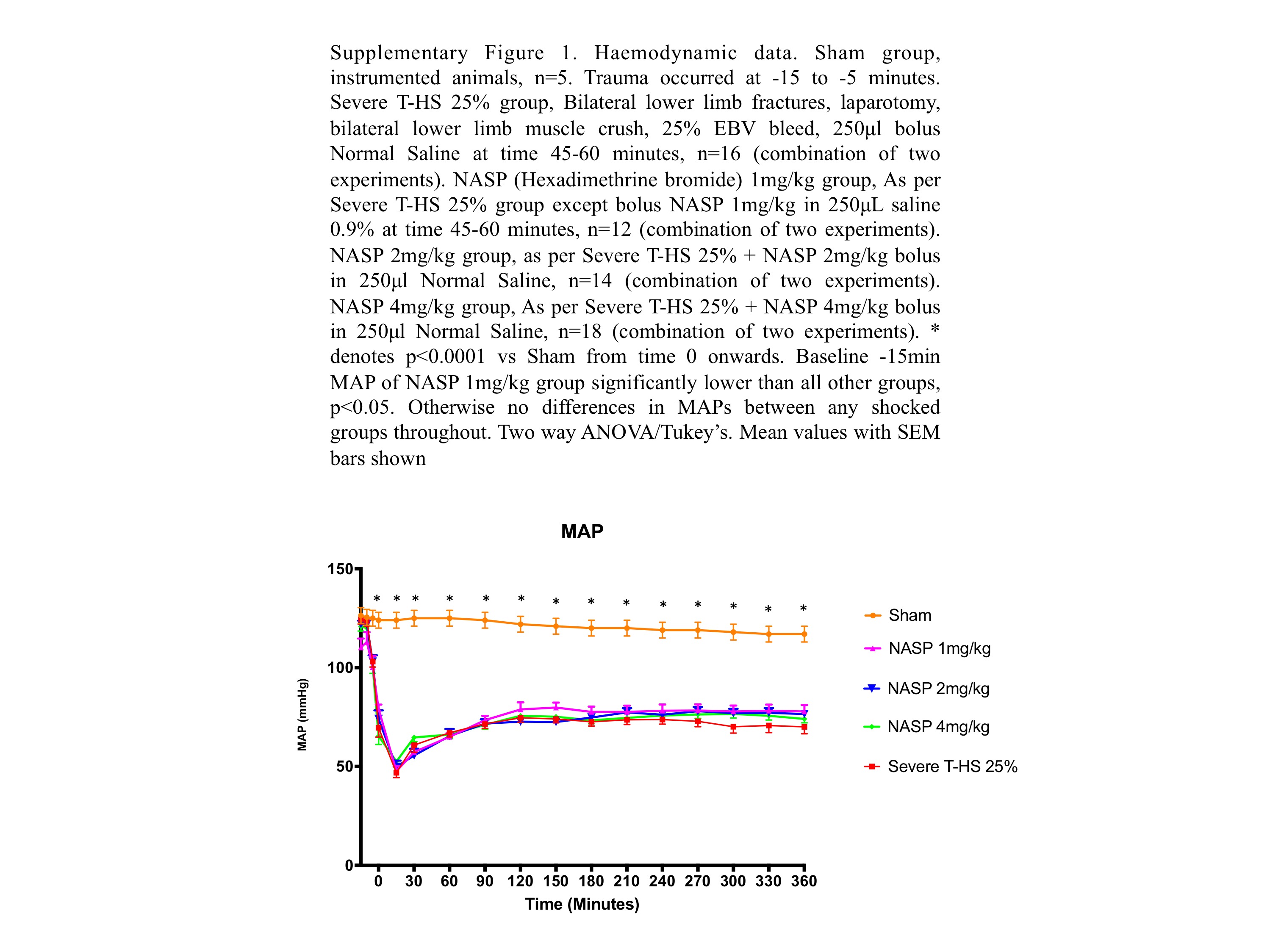

Supplement: Supplementary file 1 [file Image_1.jpg]

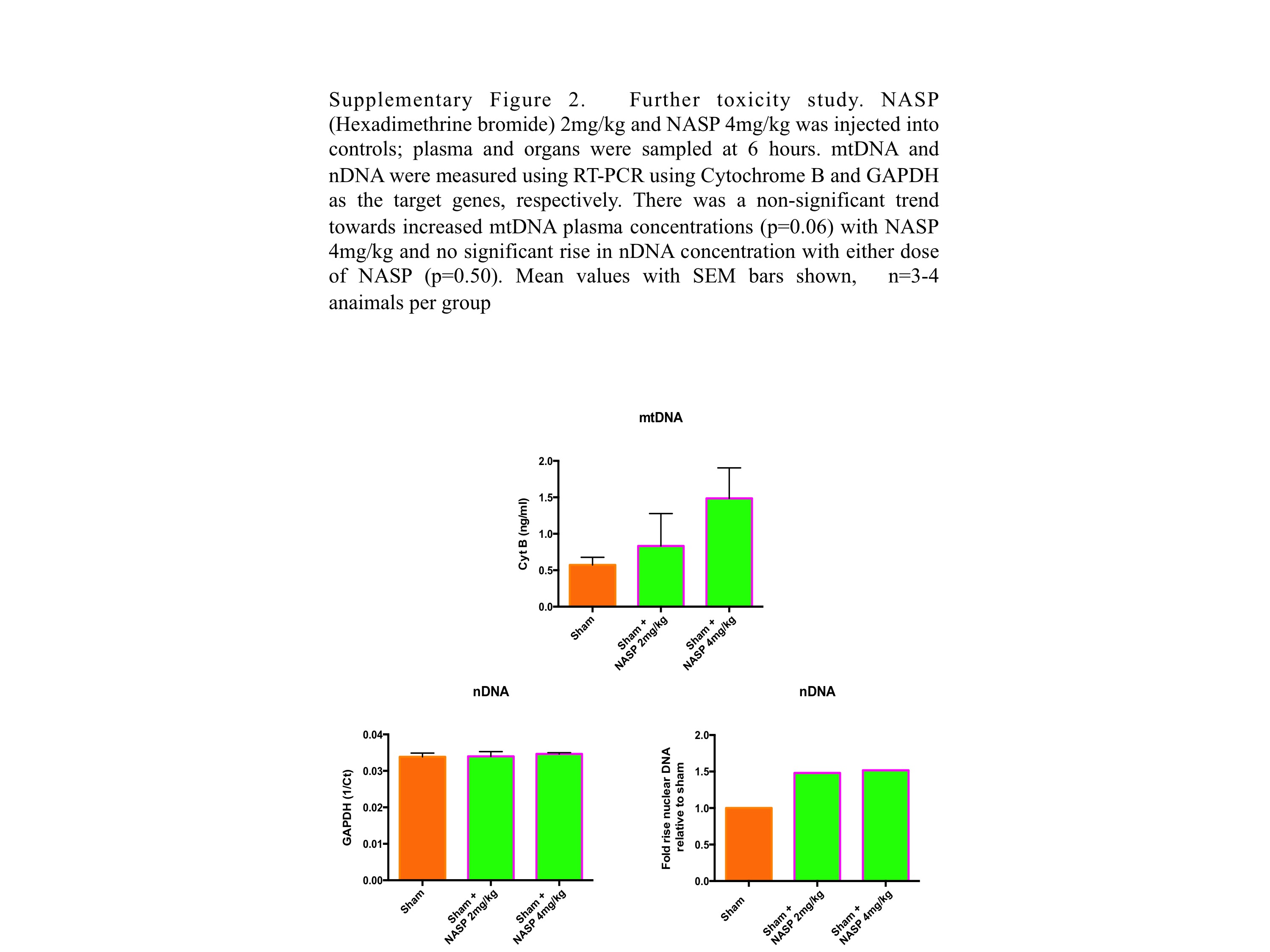

Supplement: Supplementary file 2 [file Image_2.jpg]

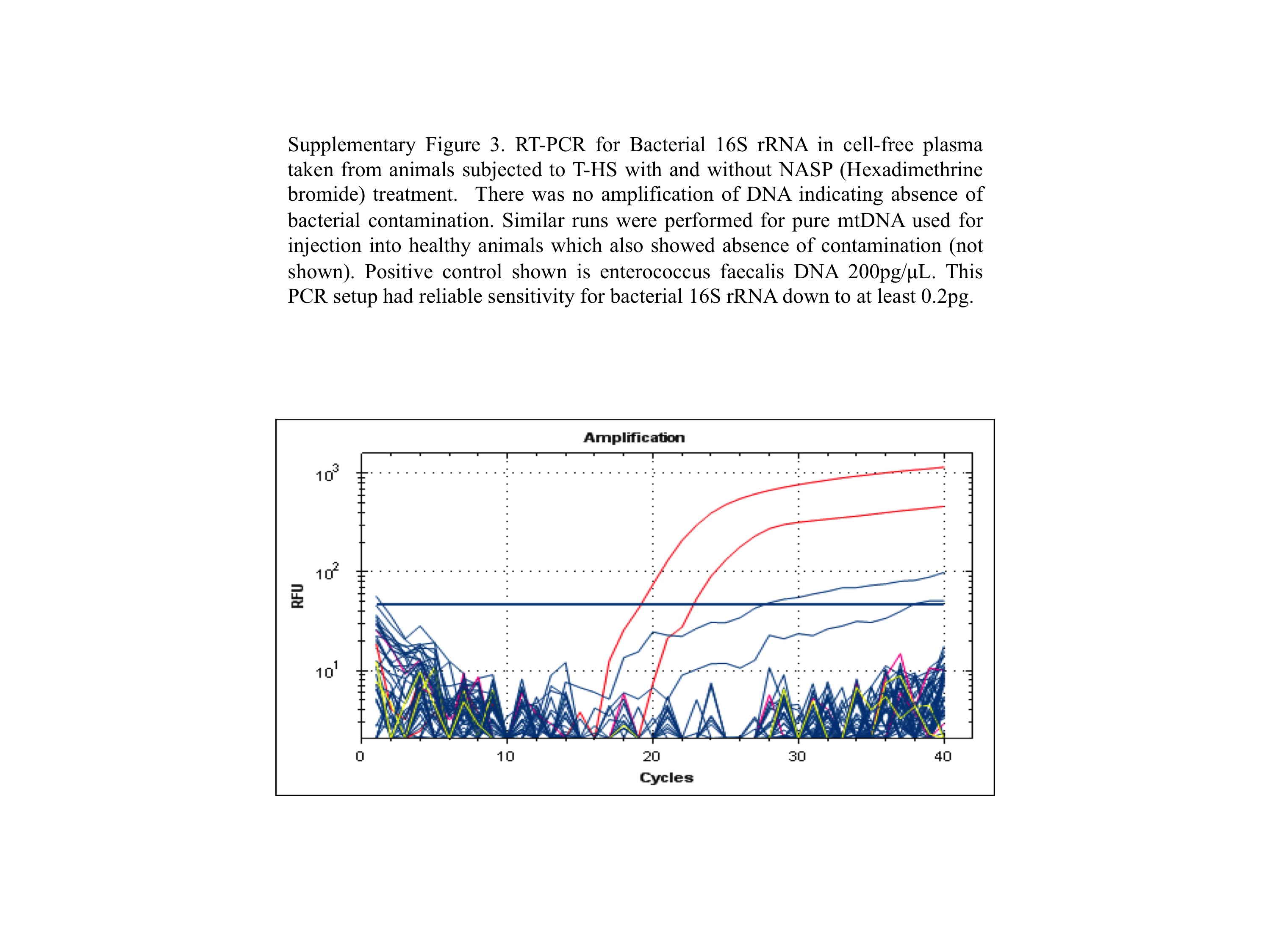

Supplement: Supplementary file 3 [file Image_3.jpg]
