# Supplementary material for: Scavenging Circulating Mitochondrial DNA as a Potential Therapeutic Option for Multiple Organ Dysfunction in Trauma Hemorrhage
Source: Front Immunol. 2018 May 8;9:891. doi: 10.3389/fimmu.2018.00891 (PMC5951958; doi:10.3389/fimmu.2018.00891)
Supplement: Supplementary file 4 [file Table_1.docx]

Supplementary Table 1: Analysis of 139 patients according to the presence or absence of MODS. These patients were picked at random from the first 367 patients recruited into the ACIT study over the period 2008-2009. The ‘Control’ group was arbitrarily defined as a low/minimal ISS with normal physiological parameters on admission to the ED. It can be seen that the development of MODS is preceded by a high ISS (particularly affecting the head and thorax) and the presence of shock on arrival to the ED. It is also more common in the elderly and in the presence of a blunt mechanism of injury. The resultant outcomes in the presence of MODS are much worse, with longer LOS, higher rates of nosocomial infection and higher 28-day mortality.

|  | **Control** | **Died <48h** | **NO MODS** | **MODS** | **p value** |
| --- | --- | --- | --- | --- | --- |
| **Demographics** |  |  |  |  |  |
| n | 16 | 11 | 85 | 27 | - |
| male (%) | 75 | 73 | 82 | 93 | 0.03 |
| Age ^ | 30 (24-40) | 31 (24-45) | 30 (22-44) | 56 (33-67) | < 0.01 |
| **Injuries** |  |  |  |  |  |
| ISS † | 4 (1-4) | 30 (25-42) | 12 (9-26) | 29 (25-36) | < 0.01 |
| Blunt (%) | 88 | 91 | 26 | 96 | < 0.01 |
| Time from injury (mins) | 72 (65-92) | 107 (96-114) | 86 (73-107) | 116 (104-120) | < 0.01 |
| GCS at scene † | 15 (15-15) | 5 (3-11) | 15 (14-15) | 14 (9-15) | < 0.01 |
| SBP in ED † | 139 (123-150) | 101 (87-136) | 136 (117-150) | 120 (96-148) | 0.03 |
| BD in ED † | 0.4 (-0.6 - 1.3) | 8.0 (6.2-12.5) | 2.0 (0.3-3.3) | 5.0 (2.3-9.0) | < 0.01 |
| Lactate in ED † | 1.2 (0.9-2.1) | 3.1 (2.3-9.5) | 2.0 (1.4-3.0) | 3.1 (1.4-4.5) | 0.06 |
| CSL † | 0 (0-0) | 1000 (375-2000) | 0 (0-250) | 500 (250-925) | < 0.01 |
| AIS Head † | 0 (0-0) | 3 (0-5) | 0 (0-1) | 2 (0-4) | < 0.01 |
| AIS Face † | 0 (0-0) | 0 (0-0) | 0 (0-0) | 0 (0-0) | 0.56 |
| AIS Thorax † | 0 (0-0) | 4 (2-5) | 2 (0-3) | 4 (0-5) | 0.01 |
| AIS Abdo & Pelvis † | 0 (0-0) | 0 (0-1) | 0 (0-0) | 0 (0-2) | 0.25 |
| AIS Extremity & Pelvis † | 0 (0-2) | 1 (0-3) | 2 (0-30) | 3 (1-3) | 0.08 |
| **Immune response** |  |  |  |  |  |
| IL-6 pg/ml ‡ | 21 (8-34) | 590 (129-827) | 70 (44-97) | 434 (220-649) | < 0.01 |
| **Outcomes** |  |  |  |  |  |
| Length of stay † | 4 (1-9) | <48h | 8 (3-20) | 18 (10-27) | < 0.01 |
| Acute Lung Injury <24h(%) | 0 | 100 | 11 | 85 | < 0.01 |
| Acute Lung Injury >48h (%) | 0 | - | 1 | 93 | < 0.01 |
| Mortality (%) | 0 | 100 | 0 | 22 | < 0.01 |
| Infections (%) | 0 | - | 8 | 67 | < 0.01 |
| ^ = Mean (SD), † median (IQR), ‡ mean (95% CI), ISS = Injury severity score, AIS = Abbreviated injury severity score, CSL = crystalloid fluid administered prior to blood draw. p value compares No MODS and MODS groups. | | | | | |
