# Supplementary material for: Scavenging Circulating Mitochondrial DNA as a Potential Therapeutic Option for Multiple Organ Dysfunction in Trauma Hemorrhage
Source: Front Immunol. 2018 May 8;9:891. doi: 10.3389/fimmu.2018.00891 (PMC5951958; doi:10.3389/fimmu.2018.00891)
Supplement: Supplementary file 5 [file Table_2.docx]

Supplementary Table 2. Pearson Correlation table of all measured variables relating to the rodent model of T-HS. Top grid indicates correlation significance, * denotes p<0.05, **** denotes p<0.0001. Bottom grid indicates correlation coefficients.

|  | **LUNG**  **MPO** | **LUNG**  **IL-6** | **PLASMA**  **IL-6** | **UREA** | **CREAT** | **ALT** | **AST** | **CK** | **nDNA** |
| --- | --- | --- | --- | --- | --- | --- | --- | --- | --- |
| **mtDNA** | ***  0.0001 | *  0.0436 | **  0.00756 | ****  3.251  x10**^-05^** | *  0.0312 | **  0.0097 | **  0.0097 | *  0.0197 | **  0.003 |
| **Lung**  **MPO** |  | ****  1.515  x10**^-07^** | ns  0.071 | *  0.017 | ns  0.206 | ns  0.348 | ns  0.105 | *  0.032 | ns  0.051 |
| **Lung IL-6** | 1.515  x10^-07^ |  | ****  3.969  x10**^-06^** | ns  0.92 | ns  0.15 | ns  0.565 | ns  0.329 | *  0.049 | *  0.011 |
| **Plasma**  **IL-6** | 0.0706 | 3.97  x10^-06^ |  | **  0.0019 | ****  3.182  x10**^-08^** | ****  9.282  x10**^-05^** | ****  2.794  x10**^-06^** | ****  1.668  x10**^-10^** | ****  3.054  x10**^-08^** |
| **Urea** | 0.0169 | 0.920 | 0.0018 |  | ****  6.770  x10**^-13^** | ****  1.607  x10**^-06^** | ****  2.799  x10**^-06^** | ****  6.199  x10**^-05^** | ****  4.703  x10**^-05^** |
| **Creat** | 0.206 | 0.1508 | 3.182  x10^-08^ | 6.770  x10^-13^ |  | ****  3.729  x10**^-12^** | ****  1.001  x10**^-13^** | ****  4.017  x10**^-10^** | ****  2.168  x10**^-08^** |
| **ALT** | 0.348 | 0.565 | 9.282  x10^-05^ | 1.607  x10^-06^ | 3.729  x10^-12^ |  | ****  2.792  x10**^-37^** | ****  3.034  x10**^-08^** | ****  1.181  x10**^-06^** |
| **AST** | 0.105 | 0.3287 | 2.794  x10^-06^ | 2.799  x10^-06^ | 1.0014  x10^-13^ | 2.792  x10^-37^ |  | ****  2.889  x10**^-17^** | ****  2.181  x10**^-10^** |
| **CK** | 0.032 | 0.048 | 1.669  x10^-10^ | 6.199  x10^-05^ | 4.017  x10^-10^ | 3.0346  x10^-08^ | 2.889  x10^-17^ |  | ****  2.071  x10**^-08^** |

|  | **LUNG**  **MPO** | **LUNG**  **IL-6** | **PLASMA**  **IL-6** | **UREA** | **CREAT** | **ALT** | **AST** | **CK** | **nDNA** |
| --- | --- | --- | --- | --- | --- | --- | --- | --- | --- |
| **mtDNA** | 0.54 | 0.41 | 0.33 | 0.47 | 0.25 | 0.30 | 0.32 | 0.28 | 0.34 |
| **Lung**  **MPO** |  | 0.86 | 0.28 | 0.35 | 0.19 | 0.14 | 0.24 | 0.32 | 0.30 |
| **Lung IL-6** | 0.86 |  | 0.84 | -0.02 | 0.31 | -0.13 | 0.21 | 0.42 | 0.52 |
| **Plasma**  **IL-6** | 0.28 | 0.84 |  | 0.36 | 0.60 | 0.45 | 0.55 | 0.67 | 0.62 |
| **Urea** | 0.35 | -0.02 | 0.36 |  | 0.69 | 0.50 | 0.51 | 0.43 | 0.45 |
| **Creat** | 0.19 | 0.31 | 0.60 | 0.69 |  | 0.67 | 0.73 | 0.63 | 0.59 |
| **ALT** | 0.14 | -0.13 | 0.45 | 0.50 | 0.67 |  | 0.95 | 0.57 | 0.53 |
| **AST** | 0.24 | 0.21 | 0.55 | 0.51 | 0.73 | 0.95 |  | 0.79 | 0.68 |
| **CK** | 0.32 | 0.42 | 0.67 | 0.43 | 0.63 | 0.57 | 0.79 |  | 0.60 |
